# A Simultaneous Feature Selection and Compositional Association Test for Detecting Sparse Associations in High-Dimensional Metagenomic Data

**DOI:** 10.3389/fmicb.2022.837396

**Published:** 2022-03-21

**Authors:** Andrew L. Hinton, Peter J. Mucha

**Affiliations:** ^1^Curriculum in Bioinformatics and Computational Biology, University of North Carolina, Chapel Hill, NC, United States; ^2^School of Medicine, University of North Carolina at Chapel Hill Food Allergy Initiative, Chapel Hill, NC, United States; ^3^Departments of Mathematics and Applied Physical Sciences, University of North Carolina, Chapel Hill, NC, United States; ^4^Department of Mathematics, Dartmouth College, Hanover, NH, United States

**Keywords:** microbiome association study, sparse association signals, pairwise logratios, compositional data, multivariate analysis, feature selection

## Abstract

Numerous metagenomic studies aim to discover associations between the microbial composition of an environment (e.g., gut, skin, oral) and a phenotype of interest. Multivariate analysis is often performed in these studies without critical *a priori* knowledge of which taxa are associated with the phenotype being studied. This approach typically reduces statistical power in settings where the true associations among only a few taxa are obscured by high dimensionality (i.e., sparse association signals). At the same time, low sample size and compositional sample space constraints may reduce beyond-study generalizability if not properly accounted for. To address these difficulties, we developed the Selection-Energy-Permutation (SelEnergyPerm) method, a nonparametric group association test with embedded feature selection that directly accounts for compositional constraints using parsimonious logratio signatures between taxonomic features, for characterizing and understanding alterations in microbial community structure. Simulation results show SelEnergyPerm selects small independent sets of logratios that capture strong associations in a range of scenarios. Additionally, our simulation results demonstrate SelEnergyPerm consistently detects/rejects associations in synthetic data with sparse, dense, or no association signals. We demonstrate the novel benefits of our method in four case studies utilizing publicly available 16S amplicon and whole-genome sequencing datasets. Our R implementation of Selection-Energy-Permutation, including an example demonstration and the code to generate all of the scenarios used here, is available at https://www.github.com/andrew84830813/selEnergyPermR.

## 1. Introduction

Metagenomic studies have enabled unprecedented insight into connections between microbes, their functions, and human disease (Martín et al., 2014). These insights are a direct result of rapid advances in next-generation sequencing technologies which are critical to metagenomic studies. Specifically, these technologies are leveraged in two popular approaches: 16S ribosomal rRNA amplicon (16S) and whole-genome shotgun (WGS) sequencing (Ranjan et al., 2016). Application of these approaches are widespread and have been used to study associations between the gut microbiome composition and colorectal cancer (Gopalakrishnan et al., 2018), inflammatory bowel disease, obesity (Manichanh et al., 2012), cirrhosis (Qin et al., 2014), and anxiety/depression (Foster and McVey Neufeld, 2013) in humans via the gut-brain axis, to name a few. The skin (Kong et al., 2012), oral (Dewhirst et al., 2010), and nasal microbiomes (Wilson and Hamilos, 2014) among other sites have also been studied in connection to disease onset and progression. With an increasing number of putative associations between microbial communities from various sites of the human body and disease being reported, microbial compositions are now being explored as diagnostic and screening tools (Zackular et al., 2014; Schlaberg, 2020). While exciting, appropriate statistical methods are still needed to overcome methodological challenges in these exceptional data, so that robust microbial biomarkers and true associations can be discovered among noisy high-dimensional metagenomic data, especially when sample sizes in observational studies are smaller than the number of features discovered.

Before metagenomic data can be used to test for associations, raw sequencing data must be appropriately processed. Taxonomic count tables are created by processing raw 16S or WGS sequencing data through bioinformatics pipelines such as Quantitative Insights Into Microbial Ecology (QIIME) (Caporaso et al., 2010) or mothur (Schloss Patrick et al., 2009) for amplicon sequencing data and Metagenomic Phylogenetic Analysis 2.0 (MetaPhlAn2) (Truong et al., 2015) or Kraken (Wood et al., 2019) for WGS data. Sequencing reads are assigned to taxonomic units where the resulting count tables are then used to profile and analyze the association between groups under study at various taxonomic levels (Phylum-Species). These data are often sparse and summarize the total number of reads for each taxonomic assignment within each sample. In current practice, total counts in these settings have been widely recognized as being uninformative due to limitations within sequencing technology (Gloor and Reid, 2016; Gloor et al., 2017; Weiss et al., 2017). That is, these data carry only relative information, requiring special statistical techniques and considerations. In particular, these relative data have a unit-sum simplex sample space where traditional Euclidean-based statistical methods have limited applicability due to geometrical differences between sample spaces. Ignoring these constraints has been shown to increase type I error (Weiss et al., 2017) and the chance of reporting spurious associations (Pearson, 1897), thus limiting the ability to generalize beyond studies.

A direct way to address simplex sample space constraints imposed by relative data is through a logratio transformation. Such transformations, which emerged from the statistical analysis of compositional data (Aitchison, 1982), function by mapping relative data from the unit-sum simplex to traditional Euclidean space. Importantly, logratio transformations are sub-compositionally coherent (Aitchison, 1982; Greenacre and Lewi, 2009), independent of the number of dimensions (Taxa, Operational Taxonomic Units (OTUs), etc.) observed in a cohort whereby true associations in the logratio form are preserved. This is not true for relative abundance where proportions change as new dimensions are considered, discovered, or removed. Sub-compositional coherence is of practical importance in biomedical studies where biomarker discovery, disease prediction, and beyond-study generalization are paramount. While logratio transformations are well-known and routinely applied in some fields (Pawlowsky-Glahn and Buccianti, 2011), their use in metagenomic datasets has been limited. Indeed, significant challenges exist when applying a logratio transformation to metagenomic data, including properly handling zeroes (Martın-Fernandez et al., 2003; Martın-Fernández et al., 2015), selecting and interpreting various logratio forms (Aitchison, 1982; Egozcue et al., 2003; Greenacre, 2019), and scale differences in counts (Lovell et al., 2020).

While the importance of the compositional nature of metagenomic data has recently been recognized (Gloor et al., 2017; Quinn et al., 2019), relatively few multivariate statistical methods have been developed directly for such data. The current state of the art methods for detecting differential abundance in compositional metagenomic data include ANOVA-like differential expression2 (Fernandes et al., 2014), Analysis of Compositions of Microbiomes (Mandal et al., 2015), and Analysis of Compositions of Microbiomes with Bias Correction (Lin and Peddada, 2020). However, these univariate methods, while powerful, are unable to detect multivariate structure within complex interconnected microbial communities (Layeghifard et al., 2017). In contrast, appropriate network and multivariate statistical methods—which are appropriate when there exist relationships between a set of variables (i.e., microbial composition) and two or more groups are to be analyzed—can be used to discover complicated microbial patterns, even in settings where there are significantly more variables than samples, and have better control over type I error (Obuchowski, 2005).

Currently, several multivariate statistical methods to detect between-group distributional differences or associations in metagenomic data can be used. A subset of these methods require a suitable beta diversity or between-sample distance (Euclidean, Manhattan, Mahalanobis, etc.) or dissimilarity (Bray-Curtis, weighted/unweighted Unique Fraction, Jaccard, etc.) metric be specified before analysis. Nonparametric tests such as permutational multivariate analysis of variance (PERMANOVA) (Anderson, 2017), Analysis of Similarity (ANOSIM) (Clarke, 1993), and the energy distance (Rizzo and Székely, 2016) can then be applied to test distributional differences between groups. Between-group association signals in metagenomic data may be sparse, i.e., resulting from differences among only a few features (OTUs, taxa, etc.), or they may be densely formed by differences between many features. Importantly, the above-mentioned nonparametric tests lack embedded feature selection and thus may have limited statistical power for detecting sparse signals in high-dimensional data.

Feature selection, which is essential to detecting sparse association signals in high-dimensional metagenomic data, requires sophisticated methods and care to simultaneously select features and test associations while maintaining reasonable type I error control (Lindgren et al., 1996; Baumann, 2003). Indeed, for this reason, the adaptive microbiome-based sum of powered score (aMiSPU) (Wu et al., 2016) and microbiome higher criticism analysis (MiHC) (Koh and Zhao, 2020) methods were developed to test sparse associations in ultra-high-dimensional OTU-based 16S data (without taxonomic aggregation requiring phylogenetic analysis of sequences). Unlike these methods and inspired from concepts put forth in the Direction-Projection-Permutation (DiProPerm) method for assessing statistical significances in high-dimensional settings (Wei et al., 2016), we introduce here the Selection-Energy-Permutation (SelEnergyPerm) method for testing and understanding sparse associations in both 16S and WGS data at the taxonomic level. SelEnergyPerm is the first method to our knowledge to utilize robust pairwise logratios to detect multivariate associations and understand them using parsimonious logratio signatures from all types of metagenomic data through simultaneous feature selection and association testing. Importantly, because SelEnergyPerm is a compositional data approach to multivariate association testing, our benchmarks focus on multivariate associations formed between a set of logratios rather than repeated univariate associations. We first show that our novel approach selects smaller subsets of non-redundant logratios that better maximize between-group associations when compared to other popular feature selection methods. Next, we show through an extensive simulation study using synthetic and empirical 16S/WGS data distributions that SelEnergyPerm has, on average, better combined power and false discovery control via the Matthews Correlation Coefficient (MCC) when compared to existing beta-diversity-based approaches. Finally, to demonstrate the utility of SelEnergyPerm in detecting and understanding differences between metagenomic distributions, we apply our method in four unique case studies utilizing publicly available metagenomic datasets where we test associations between: (1) cerebrospinal fluid microbiomes and post-infectious hydrocephalus in Ugandan infants, (2) delivery mode and the composition of infant gut microbiomes over the first 3 months of life, (3) adult gut microbiomes and abnormal fecal calprotectin levels, and (4) the gut microbiome composition of infants within the first 6 months of life and future food allergy to egg, milk, or peanuts. Notably, the case studies considered here to demonstrate SelEnergyPerm identify associations not previously reported in the original studies.

## 2. Methods

### 2.1. Selection-Energy-Permutation (SelEnergyPerm) for Simultaneous Feature Selection and Group Association Testing in Sparse High-Dimensional Compositional Data

In this section, we explain the SelEnergyPerm framework in detail. First, we describe our Differential Compositional Variation (DCV) scoring measure applied to each element of the full set of pairwise logratios (PLR) and then detail the construction of the weighted DCV network representations of these quantities. We next discuss the removal of redundant ratios using a maximum spanning tree that simultaneously maximizes logratio variance. After this, we introduce our network-based approach to feature selection and the two multivariate test statistics utilized to measure the strength of the association. We then detail our between-group association maximization algorithm with pseudocode. Finally, we describe the approach for assessing statistical significance via permutation testing using Monte Carlo sampling.

#### 2.1.1. Differential Compositional Variation Scoring

For a given metagenomic study, let **M** ∈ ℝ^*n*×*d*^ be the taxa count table for n samples and d taxa. Before working in the set of all p=(d2)=d(d-1)/2 PLR of M (up to a sign, that is, since log(*a*/*b*) = −log(*b*/*a*), we only include one ratio between each pair of taxa), we must first address the problem of zero counts. While there are numerous strategies with various drawbacks to model and impute zeros based on type/cause (Martın-Fernández et al., 2015; Palarea-Albaladejo and Martín-Fernández, 2015), there is in general no consensus on which strategy should be used in metagenomic data. Notwithstanding, here we treat zero taxa counts as being below the detection level, and we adopt a corresponding multiplicative replacement strategy for imputing zeros proposed in Martın-Fernández et al. (2015) that preserves the essential logratio and covariance structure. Specifically, we apply the closure operator to **M** to map the count data onto the unit-sum simplex, defining the matrix **X** with elements *x*_*ij*_ as


(1) $$x_{ij}={\left(C[\text{M}]\right)}_{ij}=\frac{m_{ij}}{\sum_{k=1}^{d}m_{ik}}.$$

Importantly, we set δ to be a constant equal to the smallest nonzero value across all **X** and then replace zeros to obtain **R** with elements


(2) $$r_{ij}=\{\begin{array}{cc} \delta , & x_{ij}=0\\ x_{ij}[1-\sum_{k}\delta 1(x_{ik}=0)], & x_{ij}>0 \end{array}\text{ for }i=1,\ldots ,n,$$

where **1**(*x*_*ik*_ = 0) indicates (= 1) if the element *x*_*kl*_ = 0 (and = 0 otherwise). In this way, the interpretation of zeroes is consistent across samples which may not be the case strictly following the Bayesian approach. Additionally, to limit rare taxa, we remove sparse features with a default 10% threshold. That is, we retain only those taxa present (counts ≥ 1) in at least 10% of samples. We then compute all PLRs from **R** to obtain **Z** ∈ ℝ^*n*×*p*^ including all *p* PLRs. To express the PLR transformation, we first define a PLR matrix **P**^*i*^ ∈ ℝ^*p*×*p*^ for the *i*th sample from logratios of the elements in the *i*th row of **R**, according to $p_{jk}^{i}=\log\frac{r_{ij}}{r_{ik}}$. Note that **P**^*i*^ is antisymmetric by construction, requiring only the lower or upper off-diagonals be computed to define the full frame of PLRs. We then obtain the *i*th row (denoted here as **z**_*i*_) of **Z** by reshaping the lower off-diagonal elements of **P**^*i*^ into a row vector, that is,


(3) $$\begin{array}{l} z_{i}=[p_{21}^{i},\ldots ,p_{jk}^{i},\ldots ,p_{d(d-1)}^{i}]\text{ for all }(j=2,\cdots ,d);\\ \;(k=1,\cdots ,d-1)\text{ such that }j>k. \end{array}$$

Because feature selection is critical to maximizing power and identifying sparse signals hidden within noisy high-dimensional data, we seek to reduce the dimensionality through feature selection. Notably, this setting is distinct from traditional logratio analysis (Aitchison, 1982) where dimensionality reduction using PCA is applied to all PLR transformed features to reduce dimensionality. Importantly, the set of *p* different PLRs are not independent of one another and require careful treatment to select ratios that are independent of each other. Here we propose Differential Compositional Variation (DCV), a scoring measure that enables efficient screening and ranking of PLR features within compositional data. Like the screening concept in Fan and Lv (2008) for ultra-high-dimensional feature spaces, DCV is motivated by Aitchison's compositional variation array (Aitchison, 1982) where patterns of compositional variability for a group of data can be expressed in terms of the logratio means ξ_*j*_ = *E*[Z_*j_] and variances τ_*j*_ = var[Z_*j_] where *j* = 1, …, *p*. Similarly, let ζ_*j*_ = median[Z_*j_].

The DCV score utilizes 5 different statistics to score the contained variation of each logratio; each component of DCV provides unique insight, enabling efficient screening of uninformative logratios for downstream multivariate analysis. Let **y** contain the labels for the binary classes/groups *c*_1_ and *c*_2_ under consideration, with *n*_*c*_ indicating the number of samples in class *c*. In terms of ξ_*j*_ and τ_*j*_, the first component of DCV, which measures differences in group means, is Welch's t-statistic:


(4) $$\begin{array}{l} \Delta_{j}^{1}=\frac{\xi_{j}^{c_{1}}-\xi_{j}^{c_{2}}}{\sqrt{\frac{1}{n_{1}}\tau_{j}^{c_{1}}+\frac{1}{n_{2}}\tau_{j}^{c_{2}}}}, \end{array}$$

where superscripts on $\xi_{j}^{c}$ and $\tau_{j}^{c}$ indicate the mean and variance, respectively, are computed over samples in class *c*, and we use superscripts on Δ to indicate the different components of DCV (not powers).

Next, we decompose the compositional variability of each Z_*,*j*_ using the classical F-statistic to again measure differences of means:


(5) $$\begin{array}{l} \Delta_{j}^{2}=\frac{n_{1}{\left(\xi_{j}^{c_{1}}-\xi_{j}\right)}^{2}+n_{2}{\left(\xi_{j}^{c_{2}}-\xi_{j}\right)}^{2}}{\tau_{j}^{c_{1}}+\tau_{j}^{c_{2}}}. \end{array}$$

The third component of DCV is the Brown-Forsythe F-Statistic, measuring heterogeneity of variances, computed as follows. We collect the values for the *j*th logratio in the array *a*_*ci*_, indexed as the *i*th sample in class *c*. From this, we let *b*_*ci*_ = |*a*_*ci*_ − ζ_*c*_|, where ζ_*c*_ indicates the median of class *c*, and define


(6) $$\Delta_{j}^{3}=\frac{\sum_{c}n_{c}{\left({\bar{b}}_{c\cdot }-{\bar{b}}_{\cdot \cdot }\right)}^{2}}{\sum_{c}\sum_{i}{\left(b_{ci}-{\bar{b}}_{c\cdot }\right)}^{2}/\sum_{c}\left(n_{c}-1\right)},$$

where ${\bar{b}}_{c\cdot }$ indicates the group means and ${\bar{b}}_{\cdot \cdot }$ is the overall mean of the *b*_*ci*_ values.

For the fourth component, we first define the empirical distribution function for each ordered logratio, notated simply here for the *j*th logratio of the *c*th class as


(7) $$\begin{array}{l} F_{j}^{c}\left(x\right)=\frac{1}{n_{c}}\underset{i}{\sum }1_{c}\left(y_{i}\right)1\left(Z_{ij}<x\right) \end{array}$$

where the **1**_*c*_(*y*) indicator selects out samples in class *c* and the second indicator indicates whether the *Z*_*ij*_ logratio value is less than *x*, with the sum thus counting the number of samples that satisfy both criteria. We then set the fourth component of DCV to be equal to the Kolmogorov–Smirnov statistic between the different empirical distributions for the *j* logratio:


(8) $$\begin{array}{l} \Delta_{j}^{4}=\underset{x}{sup}|F_{j}^{c_{1}}\left(x\right)-F_{j}^{c_{2}}\left(x\right)|. \end{array}$$

The fifth component of DCV measures the importance of the logratios as attributes in terms of an entropy reduction when splitting by class, as implemented using the information_gain function in the R FSelectorRcpp package (Zawadzki and Kosinski, 2021) with default settings on the logratio attributes and class response variable. The scores output from this function are organized into $\Delta_{j}^{5}$.

We aggregate the different components into the DCV matrix (logratios by DCV components):


(9) $$\text{V}=[\begin{array}{ccc} \Delta_{1}^{1} & \cdots & \Delta_{1}^{5}\\ \vdots & \ddots & \vdots \\ \Delta_{p}^{1} & \cdots & \Delta_{p}^{5} \end{array}].$$

To account for differences in scale between the DCV components, we *z*-score standardize each component (column) to define the standardized DCV matrix $\hat{\text{V}}$: ${\hat{v}}_{ij}=\left(v_{ij}-{\bar{v}}_{*j}\right)/SD\left(v_{*j}\right)$. The final set of DCV scores, $\overset{⌣}{\text{V}}\in {\mathbb{R}}^{p\times 1}$, which contains a score for each logratio, is then defined as


(10) $${\overset{⌣}{v}}_{j}=\sum_{k=1}^{5}{\hat{v}}_{jk}\text{ where }j=1,\ldots ,p.$$

#### 2.1.2. Construction of DCV Network and Conversion to Maximum Spanning Tree

Here we leverage the inherent network structure of logratios (Greenacre, 2019) to form our DCV network, defined as a directed graph where edges point from numerator vertices to denominator vertices. We then define $G=\left(N,E,\overset{⌣}{V}\right)$ to be the DCV network where *N* is the set of *d* taxa vertices, *E* is the edge set formed by all *p* pairwise logratios between taxa, and edge weights $\overset{⌣}{V}$ are the corresponding DCV scores in $\overset{⌣}{\text{V}}$ between classes. In the initial phase of feature selection on **Z**, we require the logratio subsets to meet three important properties: 1) explain maximum logratio variance, 2) form a linearly independent set, and 3) contain maximum total DCV among the different possible subsets that satisfy the first two properties. Notably, by construction the column rank of **Z** is (*d* − 1) and thus any single-component connected network containing all *d* taxa explains 100% of the logratio variance contained in **Z**. The second property requires the undirected version of the logratio subset to be acyclic, as may be achieved with a spanning tree. However, the number of spanning trees from *G* can be expressed by Cayley's formula: $T_{\left|N\right|}=|N{|}^{|N|-2}$. To circumvent considering this unmanageably large number of spanning trees, we utilize the weights imposed from the DCV scoring to enable efficient selection of a suitable spanning tree from *G*. In particular, the third property drives us to select a spanning tree that includes only edges (logratios) corresponding to the highest $\overset{⌣}{\text{V}}$ DCV scores, insofar as possible, to attempt to include maximum possible DCV, as described next.

We sort the logratios of $\overset{⌣}{\text{V}}$ in descending order by DCV score to form ${\overset{⌣}{\text{V}}}^{\prime }$ and retain the first set of *q* logratios that contain all *d* taxa to form ${\overset{⌣}{\text{V}}}^{\prime \prime }$. We then redefine the logratio network *G* = (*V, E, W*) where *V* is the set of *d* taxa vertices and *E* is the edge set corresponding to these *q* pairwise logratios, with edge weights *W* from the values in ${\overset{⌣}{\text{V}}}^{\prime \prime }$. In practice, we have always found that the resulting network at this stage is a single connected component—in the event that the network is not, additional logratios from ${\overset{⌣}{\text{V}}}^{\prime }$ should be added to *G* to make it connected. From *G* we compute the maximum spanning tree *G*_*MST*_ using the minimum spanning tree function in the R igraph package (Csardi and Nepusz, 2006), which uses a greedy approach known as Prim's algorithm (sometimes also as Jarník's algorithm). Specifically, to obtain a maximum spanning tree, we pass negative edge weights (−1× DCV scores) to the minimum spanning tree function. While the selected tree is not guaranteed to be unique given *G*, we note the presence of multiple equivalent candidate trees is highly unlikely for continuously-weighted graphs *G* (unlike for unweighted graphs). Further, we confirmed that repeated runs of the algorithm returned the same tree for each run. Finally, we define **Z**′ ∈ ℝ^*n*×(*d*−1)^ to be the subset of logratios corresponding to the edge set of *G*_*MST*_.

#### 2.1.3. Multivariate Test Statistics

SelEnergyPerm considers two multivariate test statistics to determine the statistical significance of retained subsets of logratios. The first multivariate test statistic, the Distance Components F-ratio (discoF) is utilized when between-group dispersion effects are not detected in **Z**′. The discoF statistic, proposed by Rizzo and Székely (2010), is like the traditional Analysis of variance ‘F' ratio (but does not follow an F-distribution) where the total dispersion is partitioned into between- and within-group components derived from an inter-sample Euclidean distance matrix computed from **Z**′. Computation of the discoF statistic is done here using the R energy package (Rizzo and Szekely, 2021). As described by Rizzo and Székely (2010), the discoF test statistic for binary groups is of the form


(11) $$\begin{array}{l} F_{n,\alpha }=\frac{S_{n,\alpha }}{W_{n,\alpha }/\left(n-2\right)} \end{array}$$

where *S*_*n*,α_ is the between-sample energy statistic, *W*_*n*,α_ is the within-sample dispersion statistic and 0 < α ≤ 2 is the exponent on the pairwise between-sample norm. See Rizzo and Székely (2010) and Rizzo and Székely (2016) for specific details on computing the between- and within-group components of the discoF statistic.

The second statistic, used by SelEnergyPerm when dispersion effects between groups are detected in **Z**′, is a scaled combined-F (*cF*) statistic which is distribution-free and attempts to jointly account for differences in both location and scale between distributions. The unscaled *cF* statistic is the sum of F-ratios obtained from PERMDISP2 with spatial medians (Anderson, 2006) and PERMANOVA (Anderson, 2017), computed using the R vegan package (Oksanen et al., 2020). We partition the variation of **Z**′ and define the unscaled combined-F statistic as


(12) $$\begin{array}{c} \tilde{cF}=F_{location}+F_{dispersion}=(\frac{SS_{\alpha }}{SS_{w}/(n-2)})+(\frac{SS_{T}}{SS_{E}/(n-2)}) \end{array}$$

where *SS*_α_ and *SS*_*T*_ are the between-group sum of squares components, and *SS*_*w*_ and *SS*_*E*_ are the within-group sum of square components of variation from the PERMANOVA (*F*_location_) and PERMDISP2 (*F*_dispersion_) procedures, respectively. See Anderson (2006) and Anderson (2017) for specific details on computing these between- and within-group components. Likewise, the scaled combined-F statistic that we use is computed in the same way but with *z*-score standardization relative to the permutation distribution. Let ***n*****F**_loc._ and ***n*****F**_disp._ be *m*-dimensional vectors of null *F*_loc._ or *F*_disp._ statistics sampled from the permutation distribution. We consider *m* = 10^6^ permutations here as a balance between computational cost and minimizing this variation of the estimate statistic. We scale ${\hat{F}}_{\text{loc}.}=\frac{F_{\text{loc}.}-E\left[n{\text{F}}_{\text{loc}.}\right]}{SD\left(n{\text{F}}_{\text{loc}.}\right)}$ and ${\hat{F}}_{\text{disp}.}=\frac{F_{\text{disp}.}-E\left[n{\text{F}}_{\text{disp}.}\right]}{SD\left(n{\text{F}}_{\text{disp}.}\right)}$ and define the scaled combined-F statistic as


(13) $$\begin{array}{l} cF={\hat{F}}_{\text{loc}.}+{\hat{F}}_{\text{disp}.}, \end{array}$$

taking care to note that *cF* is approximate and thus the estimate has variability based on the number of samples drawn from the permutation distribution.

#### 2.1.4. Association Maximization and Greedy Forward Selection

In this step, we focus on the multivariate structure formed by a subset of logratios. Specifically, we are interested in maximizing the between-group variation induced by a subset of logratios in a low-dimensional multivariate space. To find a minimal, statistically-significant subset of logratios that maximizes *F*_*n*,α_ (location effects only) or *cF* (dispersion and location effects) between classes, we utilize a greedy forward stepwise feature selection procedure (see Algorithm 1 in the Supplementary Material). This procedure is notated here as selectionEnergy().

#### 2.1.5. Association Significance Testing

To assess the statistical significance of the observed association *F*^*obs*^ = selectionEnergy(**Z**^*obs*^, **y**) we compute the null distribution by permutation testing via Monte Carlo sampling (Ernst, 2004). Letting the number of permutations be *k* and π be the set of random permutations of **y**, we obtain samples from the null distribution by **F**^*null*^ = selectionEnergy(**Z**^*obs*^, **π**). We then test if the *F*^*obs*^ is more extreme than what is expected at random given the data using the one-sided estimated *p*-value


(14) $$\hat{p}=\frac{1+\sum_{i=1}^{k}1(F^{null}>F^{obs})}{k+1}.$$

### 2.2. Simulation Strategy

We adapted several simulation settings to investigate and highlight key association detection characteristics of SelEnergyPerm when compared to ANOSIM, PERMANOVA, and the energy test. Additionally, to detect the presence of heterogeneity of multivariate dispersion between groups and understand its impact on association detection, we utilized the PERMDISP2 method as an indicator. The empirical association detection ability of each method was assessed within a binary classification framework. To do this, we measured the rate of each statistical test to correctly reject (Power) or accept (Type I Error) the null hypothesis (no difference between groups) at significance α = 0.05. Further, to truly assess detection capabilities, we presented each method with binary instances drawn from either the same (Null Case) or different (True Case) distributions for each scenario using Monte Carlo simulations. The Matthews Correlation Coefficient (MCC), which effectively summarizes the binary confusion matrix, was then used to measure the overall accuracy of each method's ability to detect associations across various simulation scenarios. MCC was computed as


(15) $$\begin{array}{l} MCC=\frac{\left(\text{TP}\right)\left(\text{TN}\right)-\left(\text{FP}\right)\left(\text{FN}\right)}{\sqrt{\left(\text{TP}+\text{FP}\right)\left(\text{TP}+\text{FN}\right)\left(\text{TN}+\text{FP}\right)\left(\text{TN}+\text{FN}\right)}} \end{array}$$

where TP = true positive (reject the null hypothesis for True Case), TN = true negative (accept null hypothesis for Null Case), FP = false positive (reject the null hypothesis for Null Case), and FN = false negative (accept null hypothesis for True Case). For each simulation scenario, we generated 100 simulated datasets with 40 samples each in class 1 and class 2 for the balanced binary design and 20/60 (class 1/2) samples for the unbalanced design. Given we rely on permutation testing for significance of all methods, we generate a common set of 150 permutations per dataset to consistently compute significance for each method across all scenarios and settings.

### 2.3. Simulation Scenarios (Synthetic Data)

For all synthetic data scenarios, we consider datasets with *d* = 50, 150, and 250 taxa, yielding a total of *p* = 1, 225, 11, 175, and 31, 125 pairwise logratios, respectively. We note, based on our experience, that the sizes *d* tested, while modest, are in general reflective of the actual number of taxa typically analyzed for 16S or WGS datasets after sparse taxa are removed. The following simulation scenarios are in our SelEnergyPermR R package available at https://github.com/andrew84830813/selEnergyPermR using the function scenarioN() where *N* = [1,5]. All synthetic scenarios are inspired by settings considered in Wei et al. (2016).

In Scenario 1, for the true case, we consider both multivariate location (in all dimensions) and dispersion effects that grow with increased numbers of dimensions. The increase in dispersion with dimension is similar to settings studied in Wei et al. (2016). Here, data from each sample are generated from the Dirichlet distribution **Dir**(α), commonly used to model compositional data whereby data are naturally constrained within the unit-sum simplex. Data from class 1 are simulated with α_1_ = 3. Data from class 2 are generated with $\alpha_{2}=\frac{3}{5}\log d$ where the log(*d*)/5 factor shifts the overall location and increases dispersion as the dimensionality increases. For the null case, data from both classes are generated from **Dir**(α_1_).

In Scenario 2, for the true case, we generate sparse count data from two Dirichlet distributions that differ in the location of the first component only and overall dispersion. To generally mimic real library size or total counts per sample, we use a negative binomial (NB) distribution to model the total counts for each sample and simulated as *C*_*i*_ ~ *NB*(*s,s*/(*s* + μ)) where *s* = 1 and μ = 10^7^. Notably, other discrete distributions can be used to achieve user specified library size characteristics. Count data for class 1 were generated by rounding *C*_*i*_·**Dir**(**α**_1_) where α_1_ elements are drawn from uniform distributions as


(16) $$\begin{array}{l} \alpha_{1}=\left(x_{1}\sim U_{\left[3000,5000\right]},x_{i\in \left[2,10\right]}\sim U_{\left[500,1500\right]},x_{i\in \left[11,d\right]}\sim U_{\left[1,5\right]}\right). \end{array}$$

Count data for class 2 were generated after rounding *C*_*i*_ · **Dir**(**α**_2_) where the **α**_2_ elements are drawn as


(17) $$\begin{array}{l} \alpha_{2}=\left(x_{1}\sim U_{\left[12500,17500\right]},x_{i\in \left[2,10\right]}\sim U_{\left[500,1500\right]},x_{i\in \left[11,d\right]}\sim U_{\left[1,5\right]}\right). \end{array}$$

Notably, we use the *x*_*i*∈[11,*d*]_ ~ *U*_[1,5]_ terms here to model random sparsity. For the null case, data from both classes are generated from *C*_*i*_ · **Dir**(**α**_1_).

In Scenario 3, for the true case, we generate compositional data with a large location effect that increases while the dispersion effects decrease with dimensionality. These settings are similar to settings considered for association benchmark comparisons in Wei et al. (2016). We simulate data from the additive logistic normal distribution on the simplex (Aitchison, 1982). To do this we first let ***S***_1_ = *N*(**μ**_1_, **Σ**_1_) and ***S***_2_ = *N*(**μ**_2_, **Σ**_2_) be samples drawn from multivariate normal distributions. We set **μ**_1_ = (0, …, 0) and $\mu_{2}=\left(1/\sqrt{d},\ldots ,1/\sqrt{d}\right)$ in the first 25% of dimensions and 0 in the remaining dimensions. The covariance structure was defined in the same way as in Wei et al. (2016) where **Σ** was defined with 1's along the main diagonal and 0.2 along the two diagonals off the main. From this, **Σ**_1_ = **Σ** + δ*I*_*d*_ and **Σ**_2_ = **Σ** + **U** + δ*I*_*d*_ where **U** ∈ ℝ^*d*×*d*^ is a matrix with $U_{\left[0,32/d^{2}\right]}$ entries and δ = |min(eigenvalues(**Σ**), eigenvalues(**Σ** + **U**))| + 0.05. Here row vectors from ***S*** represent additive logratio (ALR) vectors and are subsequently projected onto the simplex using the inverse additive logratio transformation defined in terms of the closure operator as ALR^−1^ = *C*[exp([*s*, 0])]. For the null case, data for both classes were simulated from *N*(***μ***_1_, **Σ**_1_).

In Scenario 4, for the true case, we generate compositional data with sparse location effects in the first dimension that grow stronger while dispersion effects grow weaker as the dimensionality increases. That is, ***S***_1_ = *N*(***μ***_1_, **Σ**_1_) and ***S***_2_ = *N*(***μ***_2_, **Σ**_2_) are defined as in scenario 3 except we set ***μ***_2_ to $\log\frac{d}{3}$ in the first dimensions and 0 in the remaining dimensions. The simplex projection and null case are done as described in scenario 3.

Finally, in Scenario 5 for the true case, we generate compositional data from the additive logistic normal distribution with a small location shift and large dispersion difference that increases with dimensionality. Let ***S***_1_ = *N*(***μ***_1_, **Σ**_1_) and ***S***_2_ = *N*(***μ***_2_, **Σ**_2_) be defined in as in scenario 3 except for μ_2_ set to $\frac{1}{\sqrt{n_{1}+n_{2}}}$ in all dimensions and **U** ∈ ℝ^*d*×*d*^ with entries drawn from *U*_[0,32]_. The simplex projection and null case are done as described in scenario 3.

### 2.4. Simulation Scenarios (Experimental Data)

For all experimental data scenarios, we used publicly available taxa count tables where sequencing data were already pre-processed. The following simulation scenarios are available in our SelEnergyPermR R package available at https://github.com/andrew84830813/selEnergyPermR using the functions simFromExpData.covarianceShift() or simFromExpData.largeMeanShft(). Notably, the simulation scenarios below first convert count data into compositional data represented on the unit simplex (i.e., normalized). To control simulation parameters, the compositional data are modeled using the additive logistic normal distribution (Aitchison, 1982). After adjusting the mean/covariance structures in a controlled way, the compositional data are then converted back to count data for analysis.

For general 16S data characteristics, we utilized the *ob_goodrich_results.tar.gz* dataset from the microbiomeHD database (Goodrich et al., 2014; Duvallet et al., 2017). We aggregated the taxa to the genus level (distinct genera = 247) and extracted the 428 healthy samples from the goodrich16S dataset for our 16S data simulations. For WGS data characteristics, we utilized the *ZeeviD2015* (Zeevi et al., 2015) dataset from the curatedmetagenome (Pasolli et al., 2017) database. We aggregated taxa counts by species (distinct species = 1,776) and extracted the 900 control samples for our WGS data simulations. Here we model the 16S and WGS count data using zero-inflated negative binomial (ZINB) models which have been shown to be a reasonable choice for modeling microbiome count data (Calgaro et al., 2020). ZINB models were fit to the 16S and WGS dataset described above using the ZINBWAVE R package (Risso et al., 2018) with default settings. For all experimental data scenarios, we used the fitted 16S/WGS ZINB models to simulate new samples for each dataset. That is, we first simulated 428 samples from the ZINB model for the 16S datasets or 900 samples for the WGS datasets. We then randomly select 40 samples per class for the balanced design and 20/60 (classes 1/2) samples for the unbalanced design. To reduce the presence of rare features we only retained features present in at least 15% of all samples for all datasets.

For Scenario 1, for the true case in both 16S and WGS datasets, we consider settings where the percent *P* = {5, 20, 35, 50} of dimensions with a location shift increases while the dispersion effect between classes remains fixed. To do this, we first simulate count data **M** from the ZINB model, map it onto the unit-sum simplex using Equation (1) and impute zeros to obtain **R** as in Equation (2). The ALR transformation is then applied to **R** to obtain ***A*** with elements *a*_*ij*_ = log(*r*_*ij*_/*r*_*id*_) for *j* = 1, …, (*d* − 1).

For each class we simulate data from *N*(***μ***, **Σ**) where


(18) $$\begin{array}{l} \mu =\text{E}\left[A\right]={\left(\text{E}\left[a_{*1}\right],\ldots ,\text{E}\left[a_{*d-1}\right]\right)}^{T}\text{ and }\Sigma_{ij}=\text{cov}\left[a_{*i},a_{*j}\right]. \end{array}$$

The variance (diag(**Σ**)) of each dimension is ranked in ascending order whereby ***μ*** and **Σ** are reordered accordingly to form ***μ***_*r*_ and **Σ**_*r*_. Of note, this is done to ensure the location shift occurs in features with minimal variance. We then simulate *S*_1_ from *N*(***μ***_*r*_, **Σ**_*r*_) with ***μ***_1_ and **Σ**_1_ using as above. Letting ***μ***_2_ = ***μ***_1_ we then shift the first *P*_*i*_% of dimensions of ***μ***_2_ by a factor of 1.25. From this we simulate *S*_2_ from *N*(***μ***_2_, **Σ**_1_). Finally, *S*_1_ and *S*_2_, which are in Euclidean ALR form, are mapped back to the simplex (relative abundance) using the inverse ALR transformation. For the null case, data for both classes are simulated from *N*(***μ***_*r*_, **Σ**_*r*_).

Finally, for Scenario 2, we consider settings for the true case (in both 16S and WGS datasets) with location shifts in the first 10% of dimensions that are confounded by increasing dispersion effects as the number of dimensions increase. Here we compute *S*_1_ in Euclidean ALR form as described in Scenario 1 (Experimental Data) such that *S*_1_ ~ *N*(***μ***_1_, **Σ**_1_). From this, **Σ**_*s*_1__ = **Σ**_1_ + **δ***I*_*d*_ and **Σ**_*s*_2__ = **Σ**_1_ + **T** + δ*I*_*d*_ where **T** is a *d* × *d* matrix with entries drawn from *U*_[0,_β__*i*_]_ and δ = |min(eigenvalues(**Σ**_1_), eigenvalues(**Σ**_1_ + **T**))| + 0.05. For 16S data β = (0.10, 1.40, 2.70, 4.00) and for WGS data β = (0.10, 4.07, 8.03, 12.00). Additionally, letting ***μ***_2_ = ***μ***_1_ we shift the first 10% of dimensions of ***μ***_2_ by a constant factor of 1.25 for WGS data and by a factor *F* = (1.20, 1.17, 1.13, 1.10) for 16S data. In all, the final multivariate forms are *S*_1_ ~ *N*(***μ***_1_, **Σ**_*s*_1__) and *S*_2_ ~ *N*(***μ***_2_, **Σ**_*s*_2__). These distributions, which are in ALR form, are mapped back onto the simplex using ${\text{ALR}}^{-1}\left(s_{i*}\right)=C\left[\exp\left(\left[s_{i*},0\right]\right)\right]$. Lastly, for the null case, data for both classes are simulated from *N*(***μ***_1_, **Σ**_*s*_1__).

For both scenarios, counts could alternatively be obtained via a negative binomial distribution (or other suitable discrete distribution) using a similar process as described in Scenario 2 of the Synthetic Data simulation section above.

### 2.5. Feature Selection Benchmarks

For the feature selection (FS) benchmark we used the Boruta R package (Kursa and Rudnicki, 2010) with maxRuns set to 100 and importance set to Gini for the Boruta FS. The glmnet R package (Simon et al., 2011) was used for LASSO FS where the elastic net mixing parameter α was set to 1 and λ was optimized via cross-validation. The caret R package (Kuhn, 2021) was used to implement Random Forest Recursive Feature Elimination (RFE) FS where 5-fold cross-validation was used to evaluate AUC and feature importance of sets *s* = {2^1^, 2^2^, …, 2^*n*^}, where *n* = floor(log_2_*p*). The FSelectorRcpp R package (Zawadzki and Kosinski, 2021) with default settings was used for the Information Gain Filter FS. For each Scenario (Synthetic Data), FS characteristics were evaluated on 200 synthetic datasets across feature space sizes of *p* = {1, 225, 4, 950, 11, 175, 19, 900, 31, 125} logratios. Performance characteristics considered were the number of logratios selected, logratio network clustering coefficient, and the combined-F statistic. Here we use the number of logratios selected by each method as a proxy for model complexity. Specifically, higher model complexity or the number of features retained increases the risk of overfitting and unnecessarily reduces the biological interpretation corresponding to the logratios. logratio networks were formed using the final subset selected by each method, defined as a graph where vertices represent taxa and edges connect taxa pairs to form a logratio. Redundancy in a logratio network of this type can be inferred from cycles in the network. While it does not detect all cycles, the clustering coefficient can be used here to detect cycles between three nodes (closed triangles vs. triplets). Computation of the global clustering coefficient was done using the R igraph package (Csardi and Nepusz, 2006). Finally, the *cF* statistic, measuring the strength of the overall association, was computed as in Equation (13) for each subset. All performance characteristics were evaluated in both balanced and unbalanced sampling designs. Computational time was recorded in seconds for each simulation scenario, feature space, and sample design. The recorded time represents the CPU time required by each FS method to select the final logratio subset. All computations were run on UNC–Chapel Hill's Linux-based Longleaf cluster in R parallelized with 10 cores using the foreach R package (Microsoft and Weston, 2020) with 5GB of RAM.

## 3. Results

**Figure 1:**
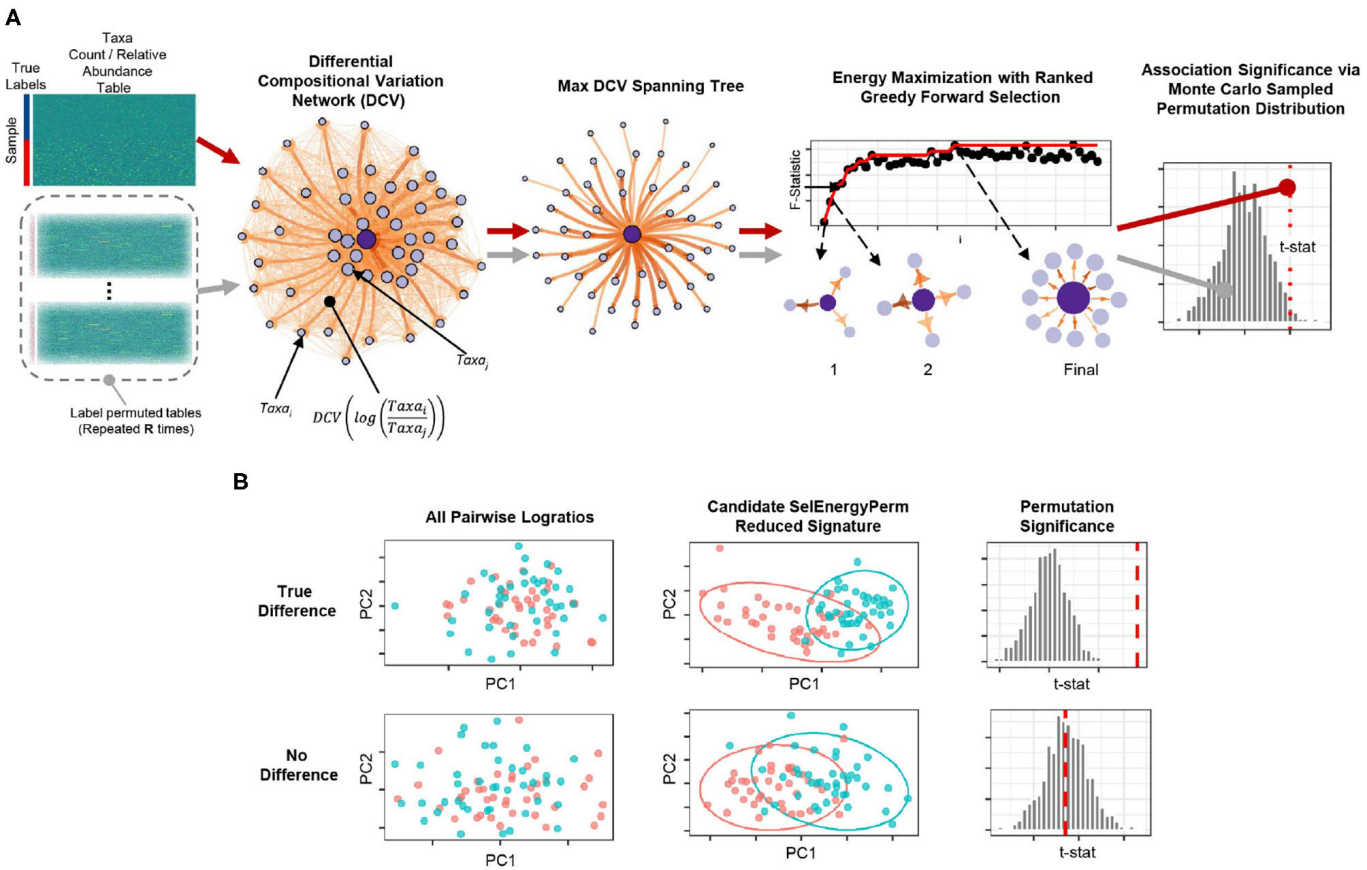
Overview of the SelEnergyPerm framework for non-parametric group association testing in metagenomic data. **(A)** Relative abundance/count data are transformed using all pairwise logratios. These logratios are subsequently scored (DCV) and used to efficiently select a subset that: (1) is independent via a maximum spanning tree and (2) maximizes the energy or association between groups via greedy optimization. The entire process is repeated using permutation testing to control false discovery and assess statistical significance. **(B)** Detection/rejection of sparse associations hidden within high dimensional data via simultaneous feature selection and permutation testing.

To robustly uncover sparse microbial signatures while simultaneously testing multivariate group associations, we based our SelEnergyPerm framework on a novel network-based feature selection approach combined with permutation testing for sparse high-dimensional low-sample-size compositional metagenomic data. Our framework (Figure 1A), which selects from all pairwise logratios between features (Taxa, OTUs, etc.), first scores the between-group variation of individual logratios using our Differential Compositional Variation (DCV) scoring measure (see Methods). From this, a weighted DCV logratio network is formed and subsequently pruned to reduce redundancy and complexity via a maximum spanning tree. Final subsets are then selected by maximizing the between-group association using a greedy forward stepwise selection procedure. Multivariate test statistics, which measure the strength of the association between groups, are then computed on the final retained subset. Statistical significance is determined by repeating this process with permuted group labels to obtain the permutation distribution of the test statistic of interest under feature selection. In this way, we determine whether the observed association is larger than what would be expected by chance (Figure 1B). To this end, our framework tests the overall null hypothesis of no association between the metagenomic composition and group labels.

### 3.1. Feature Selection Comparison to Other Methods

**Figure 2:**
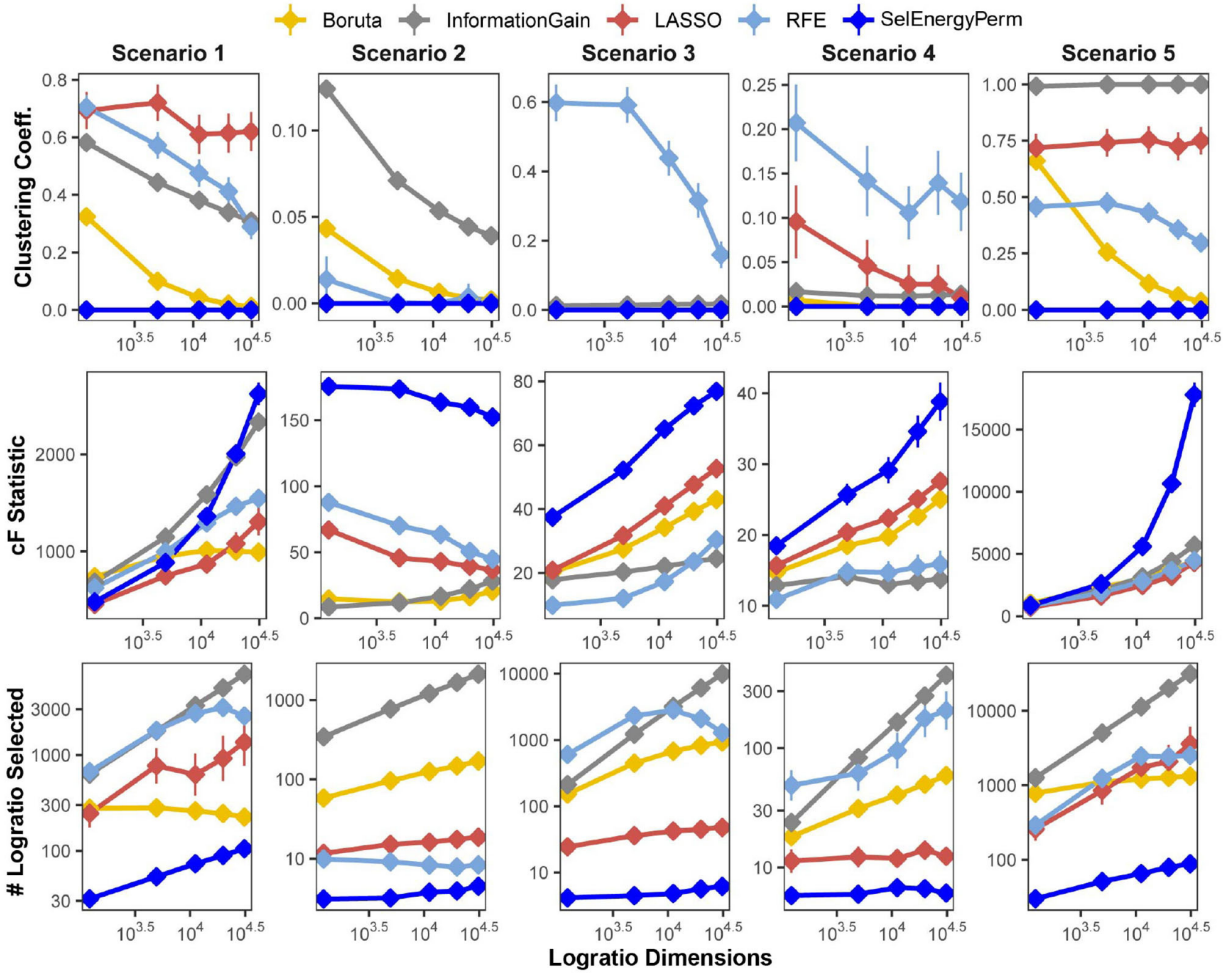
SelEnergyPerm-selected logratio subset characteristics compared with Boruta, Information Gain Filtering, LASSO, and RFE across five simulation scenarios for the balanced sampling design. Using 200 simulations for each scenario-dimension by method we assessed: (Top Row) the clustering coefficient of logratio networks formed by selected subsets returned from each method, (Middle Row) the magnitude of the association as measured by the combined-F (*cF*) statistic on selected subsets returned from each method, and (Bottom Row) the number of logratios returned by each method. Points are the mean for each experimental condition and error bars indicate 95% confidence interval.

We first benchmarked the multivariate characteristics of subsets selected by our feature selection approach against a set of other popular methods for feature selection: Boruta (Kursa et al., 2010), Least Absolute Shrinkage and Selection Operator (LASSO) (Tibshirani, 1996), Information Gain Filtering (KENT, 1983), and Random Forest Recursive Feature Elimination (RFE) (Granitto et al., 2006). The benchmarks were carried out by varying the number of logratio dimensions in the full feature set using five simulation scenarios, considering both balanced and unbalanced sampling designs (see Methods). Specifically, for subsets returned by each method, we studied the number of logratios selected (as a proxy for model complexity), the clustering coefficient of the logratio network (measuring logratio redundancy), and the combined F-statistic (strength of association, see Methods), and the computational time required to return the final subset (Supplementary Figure S1). In Figure 2, we present results from scenarios with a balanced sampling design. Notably, the results for the unbalanced sampling design scenarios are similar and do not change the overall comparative interpretation (Supplementary Figure S2). Examination of the clustering coefficient across all simulation scenarios/dimensions demonstrates that SelEnergyPerm consistently selects linearly independent subsets of logratios (Figure 2 and Supplementary Figure S2, clustering coefficient = 0), in contrast with the subsets observed in other methods tested. Of note, a clustering coefficient > 0 indicates a selected logratio subset contains at least one triple of linearly-dependent logratios (closing a triangle in the logratio network), thereby unnecessarily increasing dimensionality and model complexity. (We note that any cycle present in a logratio network indicates linear dependence, though we did not test for cycles larger than closed triangles. We emphasize that by construction the SelEnergyPerm-selected subsets do not include any such cycles). Additionally, the number of logratios retained by each method across every scenario tested revealed subsets selected by SelEnergyPerm were, on average, 14 to 149 times smaller than other methods (Figure 2 and Supplementary Figure S2).

Next, the strength of the association measured by the combined-F statistic (see Methods) indicates SelEnergyPerm-selected subsets typically capture higher between-group variations than other methods tested. In Scenarios 2–4, SelEnergyPerm subsets were observed to have on average, higher combined-F values than all other methods across all dimensions tested (Figure 2 and Supplementary Figure S2). Meanwhile, in Scenarios 1 and 5, SelEnergyPerm subsets generally performed similarly to the other methods but better as the dimensionality increased. Notably, Scenarios 1 and 5 do not simulate sparse association signals and have strong between-group dispersion effects present. These results indicate SelEnergyPerm returned subsets better capturing sparse associations (Scenarios 2–4) than the other feature selection methods tested. Computational time experiments show, across all scenarios tested, SelEnergyPerm is on average faster than Boruta and RFE but slower than LASSO and Information Gain Filtering (Supplementary Figure S1). Overall, SelEnergyPerm subsets were non-redundant, significantly more parsimonious, and captured stronger associations than other methods tested, thereby enabling robust biological interpretation using logratios in high-dimensional feature spaces.

### 3.2. Detection of Sparse Associations in Synthetic Data

**Figure 3:**
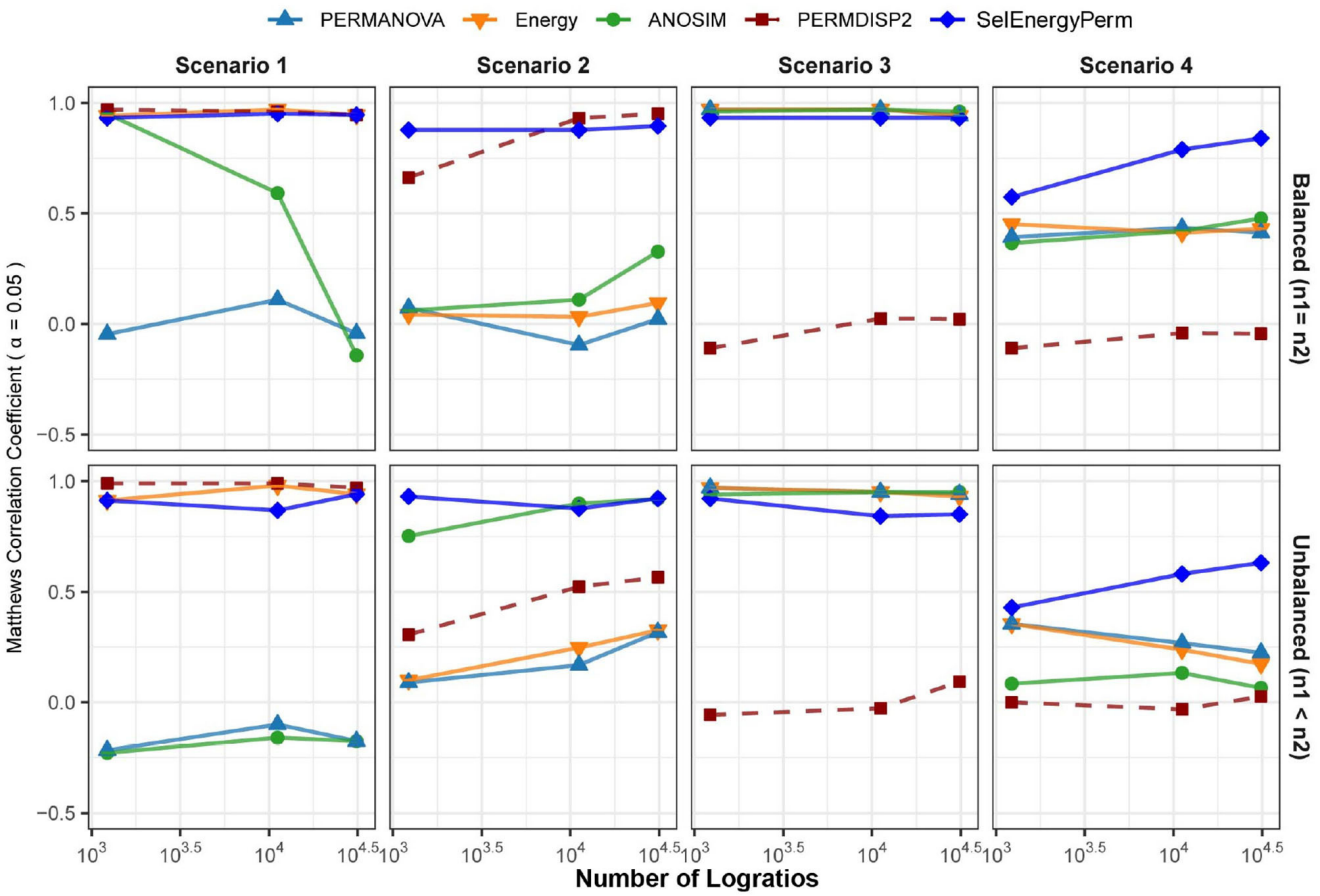
Comparison of the Matthews Correlation Coefficient measuring the ability of each method to properly detect/reject associations in data generated from synthetic distributions in both balanced and unbalanced sampling designs. For each scenario and logratio feature space size, test datasets were simulated to include data distributions that have either true between-group differences (*n* = 100) or no between-group difference (*n* = 100). Results from the PERMDISP2 procedure are displayed to indicate heterogeneity of variance between groups.

Here, we use data simulated from theoretical distributions to compare the ability of SelEnergyPerm, PERMANOVA, ANOSIM, and the energy test to detect associations in sparse high-dimensional data. That is, we are interested in determining how well each method accepts or rejects the null hypothesis (no difference between groups) when presented with two groups of data that, as ground truth, come from the same (Null Case; Type I error assessment) or different (True Case; power assessment) distributions. From this, we report the performance of each method in terms of the Matthews Correlation Coefficient (MCC) at α = 0.05 for 4 simulation scenarios (see Methods) with balanced or unbalanced sampling designs (Figure 3). For brevity, we shall refer to the collection of PERMANOVA, ANOSIM, and energy tests as the standard methods.

In Scenario 1, where data are simulated from a Dirichlet distribution with between-group location and dispersion effects that grow as the number of dimensions increase (see Methods), we see for the balanced design that both SelEnergyPerm and the energy test perform well over all dimensions (number of logratios) tested. Notably, ANOSIM loses the ability to detect associations as the number of dimensions increases while PERMANOVA performs poorly over all dimensions. The poor performance of ANOSIM and PERMANOVA is directly attributable to the underlying heterogeneity of variance present in the data generated in this scenario; these limitations of PERMANOVA and ANOSIM have been discussed previously (Anderson and Walsh, 2013). The presence of dispersion effects is confirmed with the Distance-Based Tests for Homogeneity of Multivariate Dispersions (PERMDISP2) (Anderson, 2006) method and can be observed to be steady (Figure 3-Scenario 1) and increasing across dimensions. For the unbalanced design, SelEnergyPerm and the energy test both retain strong performance and have comparable performance over most dimensions, whereas ANOSIM completely loses the ability to detect associations under the unbalanced design and PERMANOVA continues to perform poorly across all dimensions.

For Scenario 2 (Figure 3), the data distributions for each group are simulated from two Dirichlet distributions that differ in the location of the first component and overall variance. That is, this scenario embeds a sparse signal (location shift) in the first dimension with random noise in the remaining dimensions. The results for this scenario show that for the balanced case SelEnergyPerm performs significantly better than all other methods tested. For the unbalanced case, SelEnergyPerm performs better than all other methods for smaller numbers of dimensions, however, it performs similarly to ANOSIM as the number of dimensions increases. Notably, the performance of ANOSIM improves as the number of dimensions increases for both the balanced and unbalanced cases.

For Scenario 3 (Figure 3), the data distributions for the first class are simulated from the additive logistic normal distribution. Data for the second class are also generated from an additive logistic normal distribution with the same parameters (same covariance matrix) but with location shifts in the first 25% of the dimensions. Under this scenario, we observed the performance of SelEnergyPerm to be comparable to the standard methods for the balanced case and slightly worse than the standard methods for the unbalanced case. The reduced performance in the unbalanced case is attributable to the dense signal (25% of features) being in direct tension with the SelEnergyPerm objective of reduced feature selection.

Lastly, in Scenario 4 (Figure 3), a location shift only (same between-class covariance structure) was embedded in the first component of two additive logistic normal distributions, with the shift increasing with the number dimensions. Here, SelEnergyPerm outperformed the standard methods as the number of dimensions increased for both the balanced and unbalanced cases. While performing better overall relative to the standard methods, a notable decrease in performance from the balanced to the unbalanced case was observed for SelEnergyPerm. This decrease in performance was exacerbated among the standard methods where performance not only decreased between sampling designs but also generally declined as the number of dimensions increased in the unbalanced design.

Overall sparse association detection performance as measured by MCC, sensitivity, specificity, positive predictive value, negative predictive value, Youden index, and false-positive rate across all scenarios at an α = 0.05 are shown in Supplementary Figure S3. These aggregate results demonstrate SelEnergyPerm generally outperforms the standard methods for detecting sparse associations under the synthetic data simulation scenarios considered here.

### 3.3. Detection of Sparse Associations in Data Simulated From Real 16S and WGS Datasets

To further assess performance, we benchmarked our method against the standard methods on data simulated from properties observed in real metagenomic datasets. In this way, unique metagenomic data characteristics such as sparsity, over dispersion, and complex co-occurrence patterns are assessed synthetically. As above, MCC is used to assess the ability of each method to detect associations across these settings.

**Figure 4:**
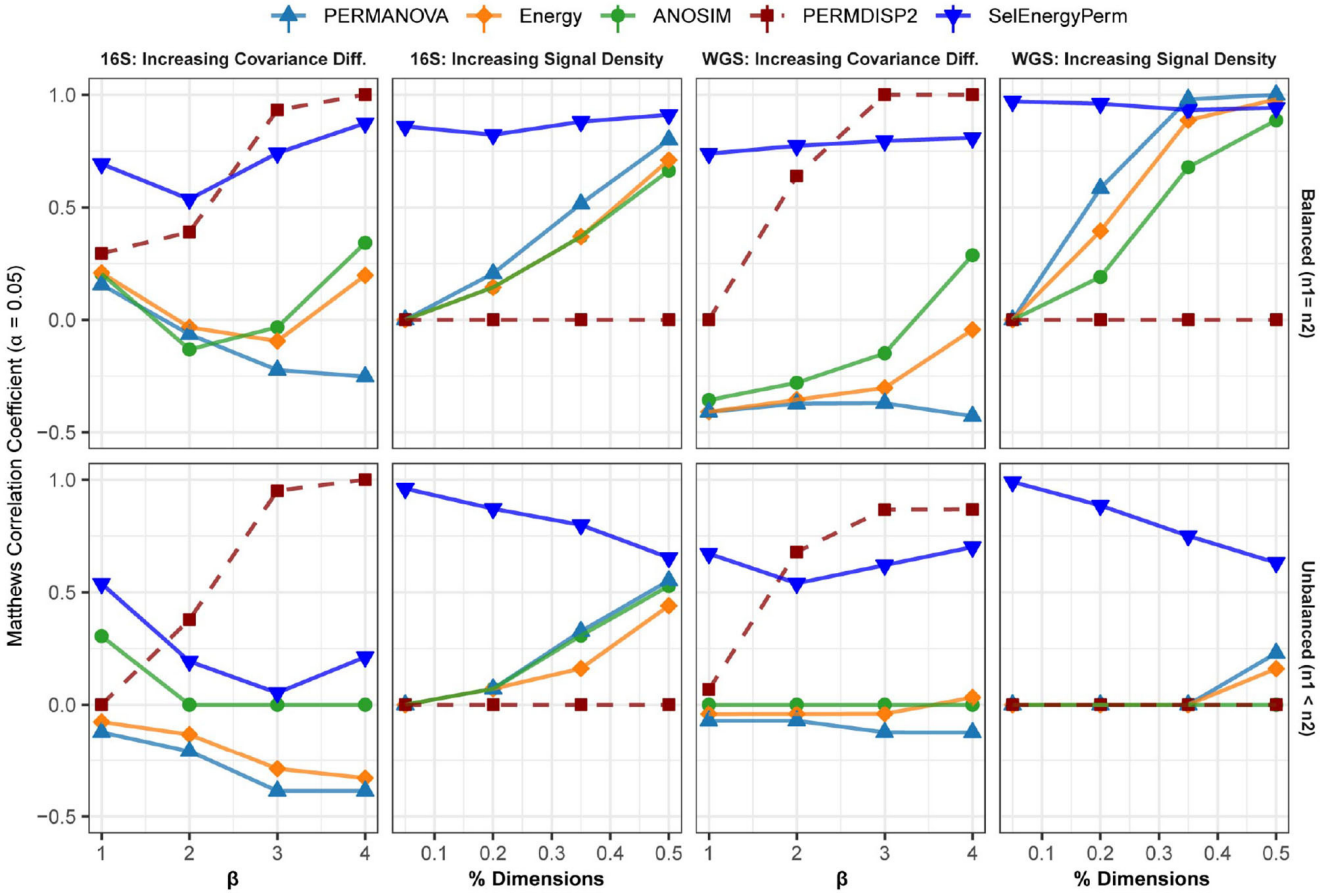
Comparison of the Matthews Correlation Coefficient measuring the ability of each method to properly detect/reject associations in data simulated from real 16S and WGS data distributions in both balanced and unbalanced sampling designs. For each data type and scenario, datasets were generated to include data distributions that have either true between-group differences (*n* = 100) or no between-group difference (*n* = 100). Results from the PERMDISP2 (dashed line) procedure are displayed to indicate heterogeneity of variance between groups.

In the first setting, (Figure 4 – 16S/WGS: Increasing Covariance Diff.), an increasing covariance effect with a decreasing location effect between classes was simulated using healthy subsets of 16S and WGS samples. The increasing dispersion effect is confirmed with PERMDISP2 for both sampling designs (Figure 4). For 16S and WGS data with a balanced sampling design, SelEnergyPerm outperforms the standard methods across all effect sizes and has strong performance as the number of dimensions increases. For 16S data with an unbalanced design, all methods performed poorly as the location shift effect increases. This trend is traceable to the strong embedded covariance effect between classes, which is a known confounder in high-dimensional association settings (Anderson and Walsh, 2013). Notably, only SelEnergyPerm and ANOSIM maintain positive MCCs on average, indicating these methods better control type I error (albeit with severely limited power) under this sampling design. For WGS data with an unbalanced design, SelEnergyPerm outperformed the standard methods and had better association detection across all effect levels.

For the second simulation setting, (Figure 4 – 16S/WGS: Increasing Location Effects), we simulated large location shifts between classes by increasing the size of the association signal from 5 to 50% of all features with fixed covariance structures. These shifts were computed using synthetic subsets of WGS and 16S samples from publicly available healthy gut microbiomes. Indeed, PERMDISP2 analysis confirmed the absence of covariance effects. For both 16S and WGS data with a balanced sampling design, SelEnergyPerm outperformed all standard methods. As expected, in both WGS and 16S data, the performance of the standard methods increased as the association signal became less sparse. Again, for the unbalanced design in both WGS and 16S data, SelEnergyPerm outperformed all standard methods. Importantly, the detection ability of the standard methods improved as the association signal became less sparse.

Finally, overall sparse association detection performance metrics are shown in Supplementary Figure S4. These aggregate results demonstrate SelEnergyPerm has better overall sparse association detection performance when compared to standard methods using data simulated from real 16S and WGS datasets.

### 3.4. Microbial Association Between Cerebrospinal Fluid Microbiomes and Post-infectious Hydrocephalus in Ugandan Infants

The cerebral spinal fluid (CSF) of Ugandan infants was profiled by Paulson et al. using 16S sequencing to characterize microbial agents associated with Post Infectious Hydrocephalus (PIH) following neonatal sepsis (Paulson et al., 2020). This processed gut microbiome dataset, retrieved from microbiomeDB (Oliveira et al., 2018), consisted of 369 distinct taxa measured on 92 samples (58 PIH and 34 Non-Post Infectious Hydrocephalus (NPIH) patients). Removing taxa not present in at least 10% of samples yielded 57 total distinct taxa (i.e., 1,596 logratios between taxa). We apply SelEnergyPerm to determine if there was an association between the microbiome composition in the CSF and PIH/NPIH disease status. We then utilize the reduced SelEnergyPerm logratio signature of PIH in CSF to gain insight into specific microbiome compositional differences.

**Figure 5:**
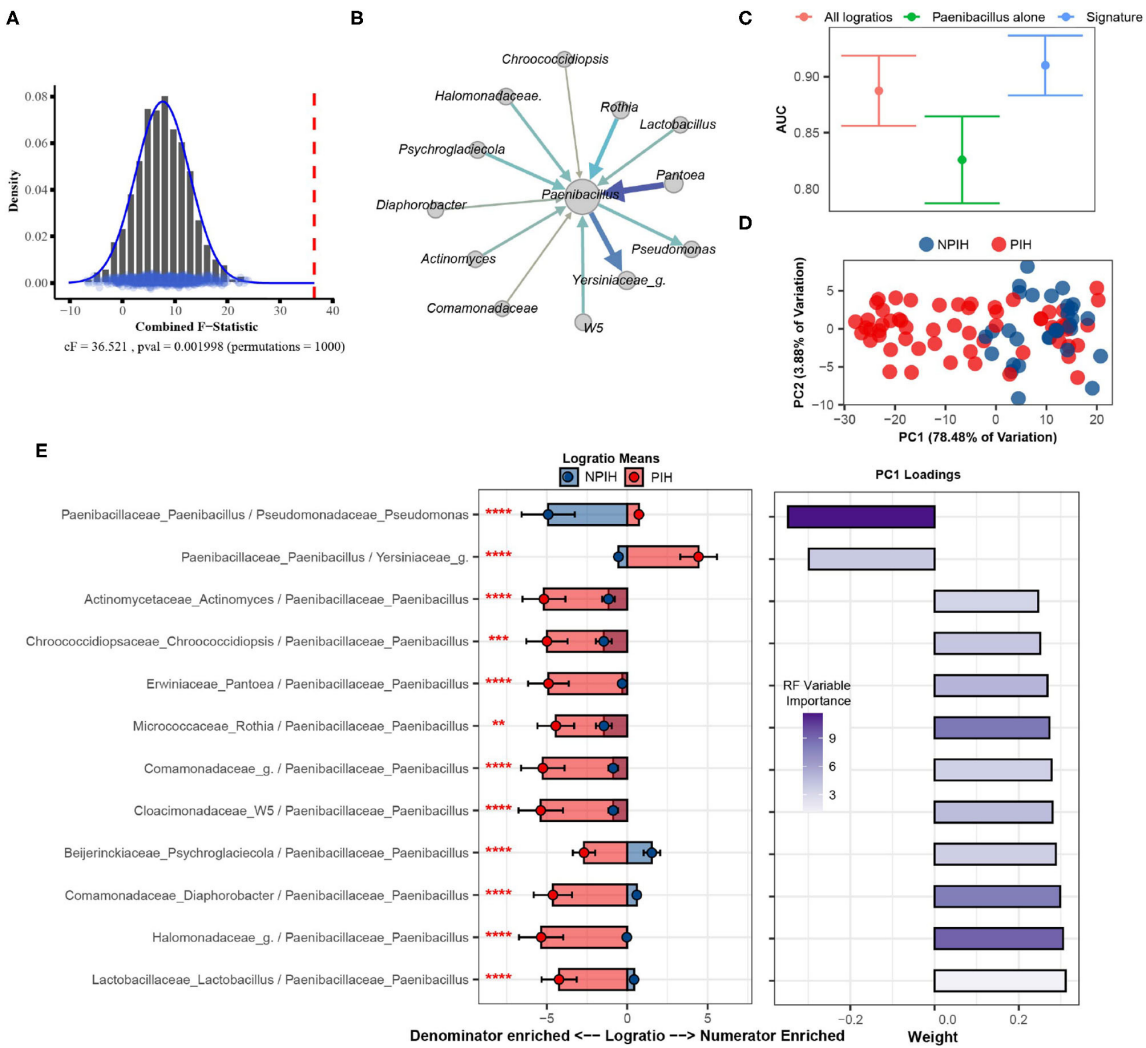
SelEnergyPerm case study examining the association between Ugandan infant's cerebrospinal fluid microbiomes and post-infectious hydrocephalus using 16S data. **(A)** SelEnergyPerm permutation test results displaying the null distribution of the *cF* statistic (Histogram, Density, and Points) and the empirical *cF* statistic (dashed red vertical line). **(B)** Random forest (RF) importance weighted directed logratio network (edges point from numerator to denominator) of the SelEnergyPerm selected signature (nodes = taxa, node size = weighted degree, edges = logratio, edge width/color = RF variable importance). **(C)** ROC (Receiver Operator Characteristic) comparisons of disease status discrimination using RF. Models were trained with repeated (*r* = 50) 10-fold cross-validation using either the SelEnergyPerm Signature, all logratios, or *Paenibacillus* alone. **(D)** Principal component analysis using the SelEnergyPerm signature. **(E)** (Left) logratio means comparison (NPIH/PIH) of each logratio included in the SelEnergyPerm signature. (Right) Loading weights of the first principal component. Significance codes (*, **, ***, ****) indicate BH corrected *p*-value < (0.05, 0.01, 0.001, 1e-4) for NPIH vs. PIH Wilcoxon Rank Sum Test. For the logratio means, positive values indicate numerator more abundant than the denominator and negative values indicate the denominator is more abundant numerator. Error bars indicate the 95% CI of the mean. Notably, error bars that do not span 0 indicate numerator/denominator is on average more abundant than the opposite.

Using SelEnergyPerm we confirm, as reported in the original study, a significant association (combined-F = 33.59817, empirical *p* = 0.007) exists between the composition of microbes in the CSF and PIH/NPIH (Figure 5A). Unlike the original study, using SelEnergyPerm we identified a multivariate association between a reduced logratio signature of 12 ratios between 13 taxa as being significantly associated with PIH/NPIH (Figure 5B). Random forest (RF) models were then used to understand the capability of this SelEnergyPerm signature for discriminating between disease statuses. Using 50 repeats of 10-fold cross-validation, we computed an Area Under the Receiver Operating Characteristic Curve (AUC) = 0.906 (0.879–0.935 95% CI) (Figure 5C). We emphasize, however, that the more complex RF models with all 1,596 pairwise logratios yielded a comparable AUC = 0.892 (0.860–0.923 95% CI) (Figure 5C). For comparison, microbiome analysis carried out in Paulson et al. revealed *Paenibacillus* alone to be important for predicting PIH; but here using only the relative abundance of *Paenibacillus* with RF we observed an AUC = 0.830 (0.792–0.867 95% CI), significantly lower than that obtained using the logratios identified by SelEnergyPerm. Combined, these results suggest the parsimonious SelEnergyPerm-derived logratio signature retains important disease interactions and better discriminates PIH vs. NPIH when compared to *Paenibacillus* alone.

To understand how the logratios in our signature work together to explain differences between the CSF microbiome of PIH vs. NPIH patients, we applied principal component analysis (PCA) (Figure 5D) and analyzed the means of the logratios. Examination of the distribution of samples shows the greatest separation between disease groups occurs along PC1 (Figure 5D), which explains 78.48% of the total variation. This separation indicates positive (negative) scores along PC1 are associated with NPIH (PIH) samples. Analyses of the logratio mean between groups for each logratio in the SelEnergyPerm signature indicate the abundance of *Paenibacillus* is significantly increased (Figure 5E) relative to taxa it is connected to (Figure 5B). Moreover, RF variable importance indicates the logratio between *Paenibacillus* relative to *Pseudomonas* to be most important for distinguishing between disease statuses. Indeed, analysis of Principal Component 1 loadings (Figure 5E) reveals increased abundance of *Pseudomonas* relative to *Paenibacillus* results in positive loadings (NPIH associated) along Principal Component 1. Overall, our results confirm, using pairwise logratios derived from SelEnergyPerm, the importance of *Paenibacillus* in PIH. Additionally, we show the interaction between the abundance of *Pseudomonas* relative to *Paenibacillus* is particularly important whereby more *Pseudomonas* is characteristic of NPIH and more *Paenibacillus* is associated with PIH.

### 3.5. Association Between Delivery Mode and the Composition of Infant Gut Microbiomes Over the First 3 Months of Life

Bokulich et al. (2016) monthly profiled the gut microbiome of infants with either a vaginal or cesarean delivery mode using 16S sequencing for the first 2 years of life. The processed dataset was retrieved from the Qiita repository using study ID 10249 (Gonzalez et al., 2018). Specifically, we extracted samples during the first 3 months of life, totaling 230 samples from 63 infants (Cesarean = 25, Vaginal = 38). We aggregated OTUs to the family-genus level which resulted in 140 distinct taxa (9,730 logratios) present in at least 10% of all samples by month. Here we apply SelEnergyPerm to determine if the gut microbiomes are different between the delivery modes of infants at any of the first 4 monthly time points collected (0–3 months). Secondarily, we studied our reduced logratio signatures to understand gut microbiome compositional differences between delivery modes at time points where significant differences were detected.

**Figure 6:**
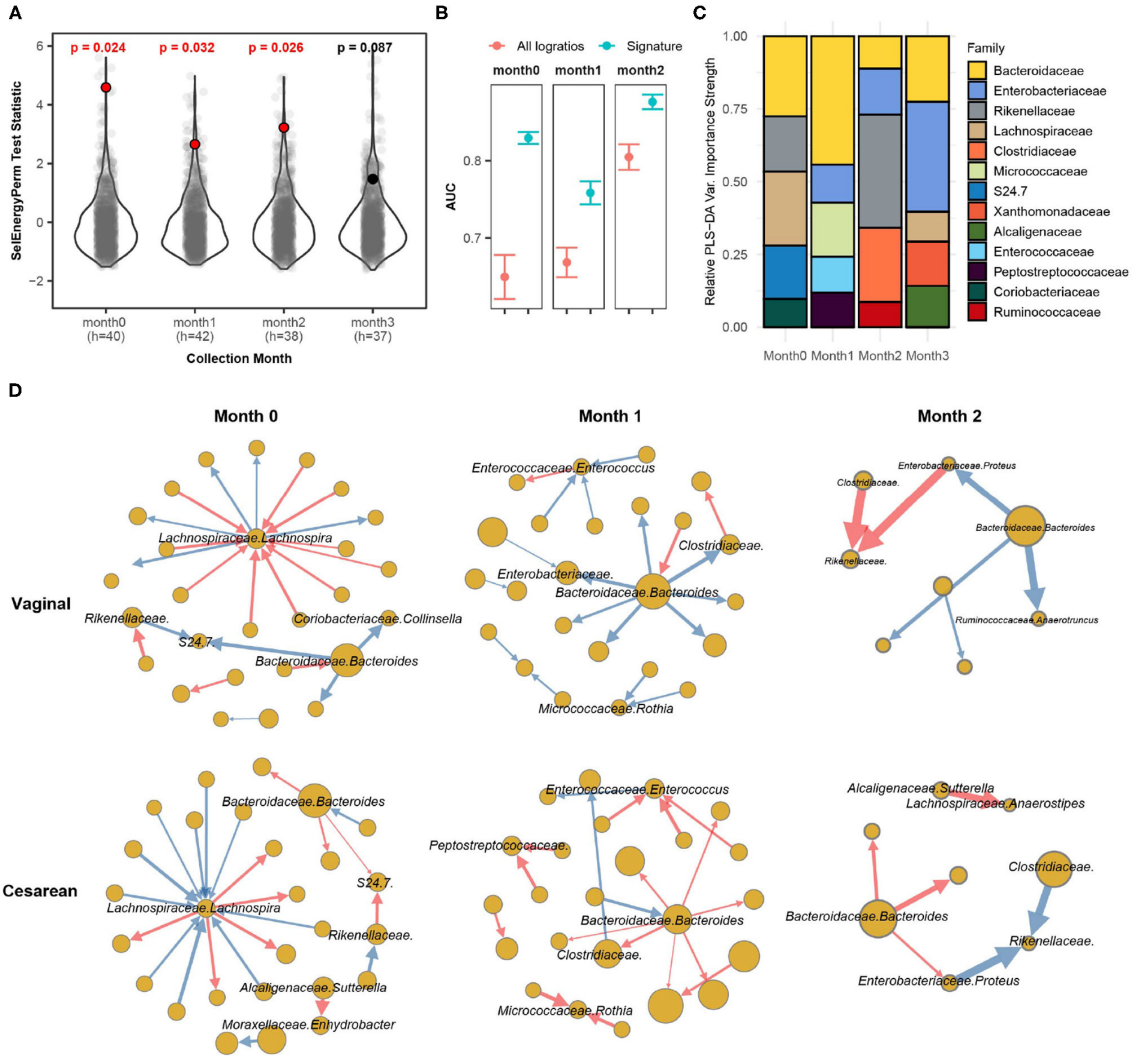
SelEnergyPerm case study examining the association between delivery mode and the gut microbiome composition of infants over the first 3 months of life using 16S data. **(A)** SelEnergyPerm permutation test (permutations = 1,000) results displaying the null distribution of the test statistic (violin and gray points) and the empirical test statistic (red if significant, black otherwise) with Benjamini-Hochberg corrected *p*-values. Test statistics values were *z*-score scaled (by Collection Month) for ease of visualization. **(B)** AUC comparisons of delivery mode discrimination using PLS-DA. Models were trained with repeated (r = 20) 5-fold stratified (delivery mode and host) cross-validation using either the SelEnergyPerm signature or all logratios. Points represent the mean AUC and error bars indicate the 95% CI. **(C)** Relative taxa strength by family measuring the importance of each taxon for discriminating between delivery modes across each collection time point. Relative strength was computed using the top 5 nodes derived from the PLS-DA variable importance weighted logratio networks across each collection time. **(D)** Directed (edges point from numerator to denominator) network of the SelEnergyPerm-derived signature by month and delivery mode weighted by the absolute logratio means [nodes = taxa, node size = mean strength, edge = logratio, edge width = logratio mean, red edges = negative logratio mean (incoming node more abundant), blue edges = positive logratio mean (outgoing node more abundant)].

Applying SelEnergyPerm to each time point with restricted permutation testing to account for repeated host microbiomes within a collection month and correcting for multiple comparisons using the Benjamini-Hochberg (BH) procedure, we found significant differences in the composition of the gut microbiomes between delivery modes during the collection periods in months 0–2 (Figure 6A). Notably, restricted permutation testing with PERMANOVA and ANOSIM using all taxa pairwise logratios (PLR) failed to detect differences between the gut microbiomes at α = 0.05. Notably, the all pairwise logratio PERMANOVA results reported here similarly fail to detect an association between delivery mode as reported in (Bokulich et al., 2016) where PERMANOVA with UniFrac distance was applied. Similarly, when using Partial Least Squares Discriminate Analysis (PLS-DA) with repeated cross-validation stratified by both delivery mode and host, we observed the AUC of the SelEnergyPerm-derived signatures to be higher across all time points compared to models trained using all PLR (Figure 6B). We next used the reduced logratio signatures and their PLS-DA variable importance scores to better understand which taxa are most important for discriminating between delivery modes. Indeed, aggregating to the family level for ease of interpretation, we found during months 0 and 1 that *Bacteroidaceae* were top contributors to compositional differences (Figure 6C). This pattern changed during month 2 where *Rikenellaceae* taxa were most important for discriminating between delivery modes (Figure 6C). Finally, to understand the direction of these differences (i.e., for a given logratio, is the numerator more abundant than denominator or vice-versa between groups), we analyze the directed logratio means network of the SelEnergyPerm signature relatively (i.e., taxa A more/less abundant than taxa B) between delivery modes (Figure 6D). Specifically, given the spoke-hub character of the observed network, with a single highly connected and central node in the directed maximum spanning tree formed by the SelEnergyPerm signature, we can see month 0 is dominated by differences between logratios that include *Lachnospira* and *Bacteroides*, which are more abundant relative to their network of taxa connections for infants with a vaginal delivery mode whereas the opposite is true for infants with a cesarean delivery mode. For month 1, *Bacteroides* are observed to be more abundant relative to its network of taxa connections for infants with a vaginal delivery mode. The opposite is true for infants with a Cesarean delivery mode where *Bacteroides* are less abundant within its network of taxa connections. Finally, for month 2, *Rikenellaceae* taxa can be observed to be more (less) abundant relative to both *Clostradiacea* and *Proteus* taxa for infants with a vaginal (Cesarean) delivery mode.

### 3.6. Association Between Abnormal Fecal Calprotectin Levels and the Composition of the Gut Microbiome in Healthy and Inflammatory Bowel Disease Individuals

Here we apply SelEnergyPerm to analyze WGS microbiome data from the integrative human microbiome project (Proctor et al., 2019), a longitudinal study designed to uncover interactions between disease and human-associated microbial communities. Specifically, using the inflammatory bowel disease (IBD) part of the integrative human microbiome project study, we tested whether there exists an association between the gut microbiome composition and abnormal levels of fecal calprotectin, a protein marker of intestinal inflammation (Proctor et al., 2019). Processed microbiome data were extracted from the Inflammatory Bowel Disease Multiomics Database (Lloyd-Price et al., 2019) resulting in 399 samples (93 individuals) reporting fecal calprotectin levels that were above 120 (abnormal; n = 190) or below 50 (normal; n = 209). There were 122 species identified (7,381 logratios) as being present in at least 10% of all samples.

**Figure 7:**
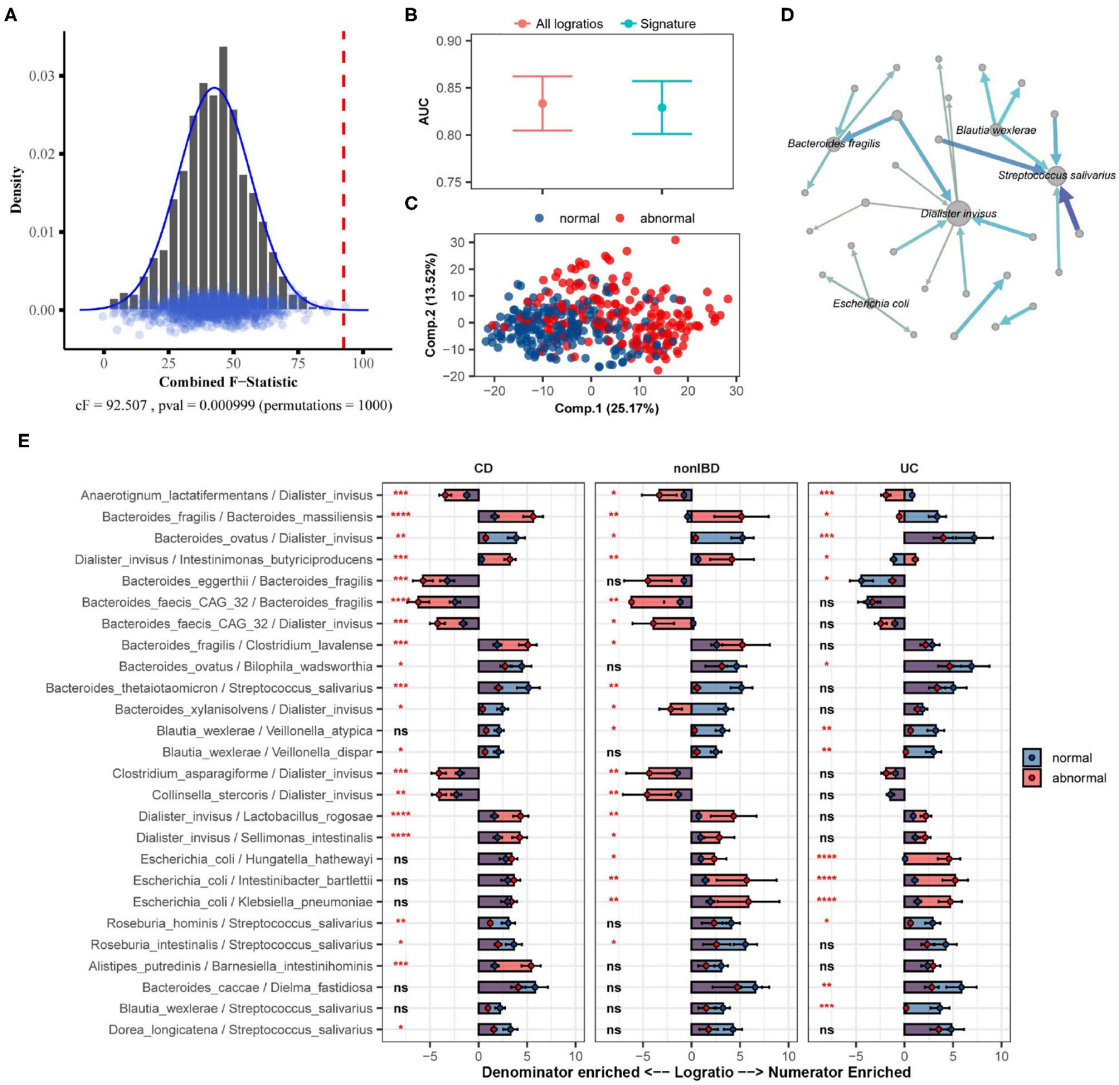
SelEnergyPerm case study examining the association between abnormal fecal calprotectin levels and the gut microbiome composition in nonIBD and IBD individuals using WGS data. **(A)** SelEnergyPerm permutation test results displaying the null distribution of the *cF* statistic (Histogram, Density, and Points) and the empirical *cF* statistic (dashed red vertical line). **(B)** AUC comparisons of fecal calprotectin level (Abnormal/Normal) discrimination using PLS-DA with 2 components. Models were trained with repeated (*r* = 20) 10-fold cross-validation using either the SelEnergyPerm signature or all logratios. Points represent the mean AUC and error bars indicate the 95% CI. **(C)** PLS-DA latent space projection plot extracted from final PLS-DA model fit using the full dataset with the SelEnergyPerm signature. Points represent non-IBD or IBD samples. **(D)** Directed network (edges point from numerator to denominator) of the SelEnergyPerm-selected logratio signature (nodes = taxa, node size = DCV strength, edges = logratio, edge width/color = PLS-DA Variable Importance). The top 5 taxa names by strength (PLS-DA Variable Importance) are displayed. **(E)** Logratio means comparison (normal/abnormal fecal calprotectin level) of each logratio included in the SelEnergyPerm signature stratified by Crohn's Disease (CD), Ulcerative Colitis (UC), and non-IBD individuals. Significance codes (ns, *, **, ***, ****) indicate BH corrected (within diagnosis) p-value < (Not Significant, 0.05, 0.01, 0.001, 1e-4, 0) for normal vs. abnormal Wilcoxon Rank Sum Test. Error bars indicate the 95% CI of the mean. Notably, error bars that do not span 0 indicate numerator/denominator is on average more abundant than the opposite.

Using restricted permutation testing, accounting for the order of visit and diagnosis of Ulcerative Colitis, Crohn's Disease, or non-IBD, SelEnergyPerm identified a significant association (combined-F = 92.507, p = 0.000999, 1,000 permutations) between the composition of the gut microbiome and abnormal levels of fecal calprotectin in corresponding stool samples (Figure 7A). Notably, both ANOSIM and PERMANOVA with restricted permutation designs using all pairwise logratios (PLR) also detected this association. To assess whether the associated SelEnergyPerm logratio signature (25 logratios between 31 species) retained enough information to adequately discriminate between levels of fecal calprotectin, we estimated the discriminatory ability both using the reduced signature and using all PLR. Using repeated cross-validation with PLS-DA we found the SelEnergyPerm signature (AUC = 0.829, 0.803–0.854 95%CI) to have comparable performance to PLS-DA models trained using all logratios (AUC = 0.833, 0.803–0.862 95%CI) (Figure 7B). Examination of the latent space projection of a final PLS-DA model fit using the SelEnergyPerm signature reveals strong separation between individuals with normal vs. abnormal fecal calprotectin levels (Figure 7C). A directed logratio network of the SelEnergyPerm signature weighted by PLS-DA variable importance shows logratios involving *Dialister invisus, Streptococcus salivarius, Bacteroides fragilis, Escherichia coli*, and *Blautia wexlerae* to be most important for discriminating between levels of fecal calprotectin (Figure 7D). Interestingly, stratifying the logratio signature by diagnosis reveals both shared (significant between diagnosis differences across all groups) and distinct (significant between diagnosis differences among a single group) gut microbiome differences (Figure 7E). Particularly increased abundance of *Dialister invisus* relative to *Bacteroides ovatus, Intestinimonas butyriciproducens*, and *Anaerotignum lactatifermentans* was observed to be associated with abnormal fecal calprotectin independent of diagnosis. Notably, the associations reported here are novel and were not reported or tested in the original study.

### 3.7. Association Between the Gut Microbiomes of Infants in Early Life and the Development of Allergen-Specific Sensitization

In this case study, we apply SelEnergyPerm to WGS gut microbiome data from the DIABIMMUNE study (Vatanen et al., 2016). The focus of this longitudinal study was to characterize interactions between the immune system and the gut microbiome in the context of autoimmunity and allergy. Specifically, the gut microbiomes of infants from Finland, Russia, and Estonia were profiled monthly during the first 3 years of life. Here we apply SelEnergyPerm to test if associations exist between allergy status and the composition of the gut microbiome at 6-month intervals during the first 2 years of life. Allergy status was defined as food allergy (FA) if the host reported an allergy to egg, peanuts, and/or milk at year 2 (non-FA otherwise). We extracted 646 samples from 192 infants (Russia = 53, Finland = 70, Estonia = 59) across 170 unique species (14,365 logratios).

**Figure 8:**
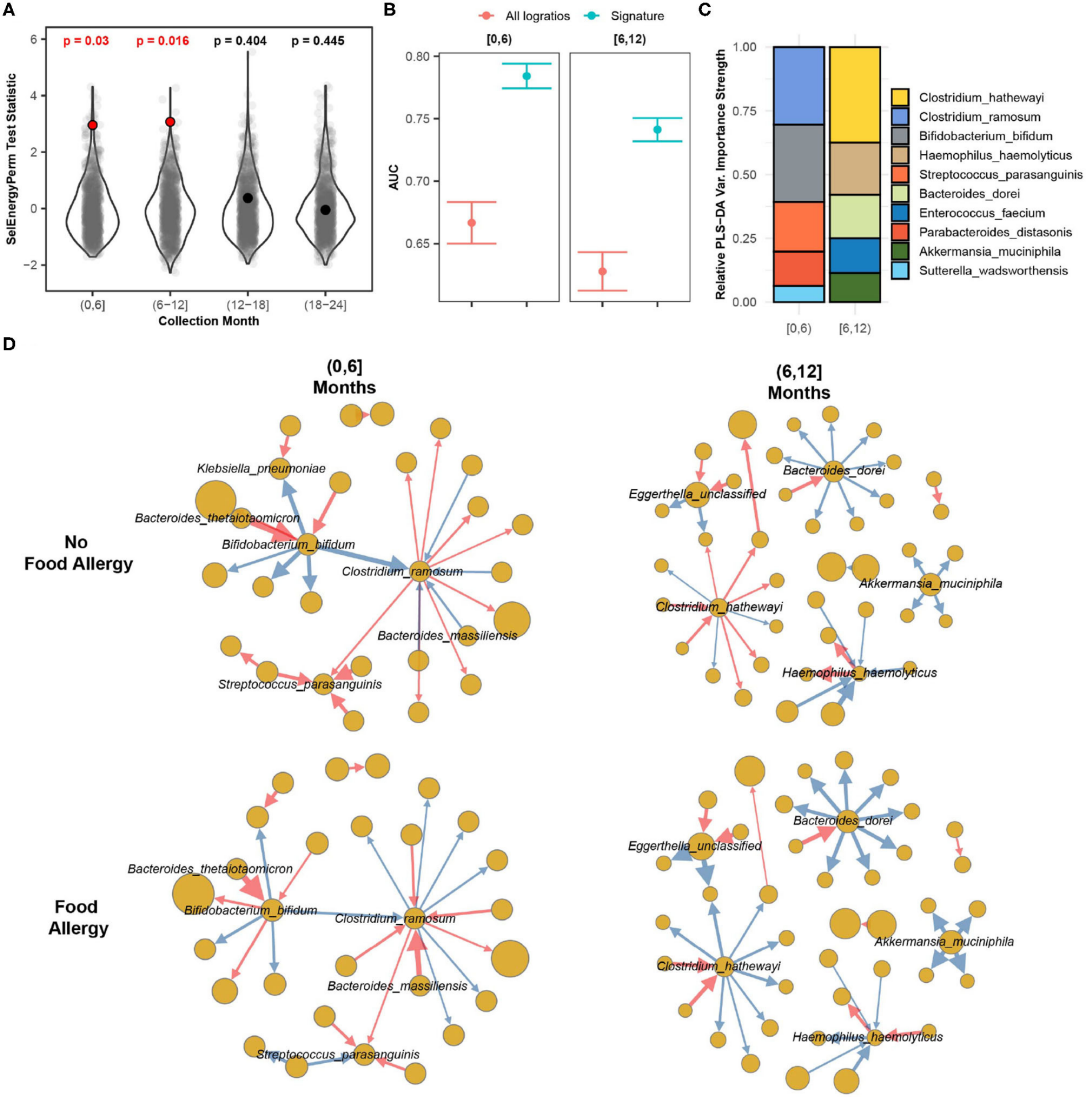
SelEnergyPerm case study examining the association between the gut microbiomes of infants in early life and the development of food allergy later in life. **(A)** SelEnergyPerm permutation test (permutations = 1,000) results displaying the null distribution of the test statistic (violin and gray points) and the empirical test statistic (red if significant, black otherwise) with Benjamini–Hochberg-corrected *p*-values. Test statistics values were *z*-score scaled by collection period to improve visualization. **(B)** AUC comparisons of future food allergy development discrimination using PLS-DA. Models were trained with repeated (*r* = 20) 10-fold stratified (host and food allergy development) cross-validation using either the SelEnergyPerm signature or all logratios. Points represent the mean AUC and error bars indicate the 95% CI. **(C)** Relative taxa strength by family measuring the importance of each taxon for discriminating between food allergy statuses later in life across each collection month. Relative strength was computed using the top 5 nodes derived from the PLS-DA variable importance weighted logratio networks across each collection month. **(D)** Directed (edges point out from numerator to denominator) networks of the SelEnergyPerm-derived signature by collection period and food allergy development weighted by the absolute logratio means [nodes = taxa, node size = mean strength, edge = logratio, edge width = logratio mean, red edges = negative logratio mean (incoming node more abundant), blue edges = positive logratio mean (outgoing node more abundant)].

Using restricted permutation testing to account for repeated host microbiomes and host country we applied SelEnergyPerm to each timeframe and corrected for multiple comparisons using the BH procedure. We found significant differences in the composition of the gut microbiomes between allergy status during both the first 6 months and the 6–12 month collection periods (Figure 8A). PERMANOVA and ANOSIM using all taxa PLR detected differences between the gut microbiome during the first 6 months of life but did not detect differences between the gut microbiomes during the remaining time frames at α = 0.05 after correcting for multiple comparisons. This difference is further apparent when comparing the discriminatory ability between the SelEnergyPerm signature and all logratios. Using Partial Least Squares Discriminate Analysis (PLS-DA) with repeated cross-validation stratified by allergy status, host, and month, we observed the AUC of the SelEnergyPerm-derived signatures to be significantly higher across all time points when compared to models trained with all logratios (Figure 8B). Using the SelEnergyPerm logratio signatures and the corresponding PLS-DA variable important scores we next examine which taxa are important for discriminating between food allergy statuses later in life. Stratifying by month and selecting the top 5 species by strength (weighted degree) from our variable importance logratio network, we found *Clostridium ramosum, Streptococcus parasanguinis*, and *Bifidobacterium bifidum* to be major contributors to the DCV score between allergy status during the first 6 months of life (Figure 8C). However, for the 6–12 month period we found the abundance of *Clostridium hathewayi, Bacteroides dorei*, and *Haemophilus haemolyticus* to be major contributors to DCV (Figure 8C). A review of the logratio mean networks (Figure 8D) between allergy status during the first 6 months shows *Clostridium ramosum* is, in general, more abundant relative to species (node strength indicated by size) it is connected to in infants with FA vs. non-FA. Further, during the 6–12 month period we see more distinct differences in the logratio mean networks whereby *Bacteroides dorei* can be observed to be more abundant relative to species it is connected to in FA infants. We also observe *Clostridium hathewayi* to be more (less) abundant than the species it is connected to in infants with FA (without FA). Notably, the associations reported here are novel and were not reported or tested in the original study.

## 4. Discussion

We here presented SelEnergyPerm, a group association testing framework for high-dimensional metagenomic data with sparse microbiome associations between groups. False discovery is properly controlled for by repeating the entire process with permuted labels using appropriate permutation test design (e.g., restricted design for longitudinal supervised data) for statistical significance (Ernst, 2004). Importantly, because multivariate effect sizes are not well studied, in case studies we use AUC as a proxy of effect size between groups (i.e., AUC = 1 indicates perfect separation, and AUC = 0.5 for no separation). Notably, AUC used in this context indicates strength of association rather than out-of-sample predictive accuracy. We also emphasize that SelEnergyPerm is designed to detect sparse associations in the sense of including relationships (logratios) between a relatively small number of taxa, whereas identifying associations in sparse taxa appearing in a relatively small number of samples would require different methods.

The association detected by the SelEnergyPerm framework is expressed as a logratio signature. That logratio signature can then be further analyzed with traditional statistical techniques to better interpret and visualize (e.g., with PCA or PLS-DA) how the microbiome is associated with the phenotype of interest. In the context of microbiome studies, each logratio represents the interaction between a pair of taxa. Rather than comparing the count (or even the count relative to the total) of each taxon separately between samples, the value of each logratio is instead compared between samples. Working in terms of logratios forces a comparison between samples that directly utilizes and respects the compositional nature of microbiome data. In particular, whether a specific taxon is “high” or “low” in a sample is not in itself meaningful, even if expressed as a fraction of total counts. In contrast, as indicated by Aitchison (1982), logratios enable robust comparisons between samples as they inherently account for variability due to, e.g., different sequencing instruments or different total reads. Moreover, in detecting sparse associations, logratios provide greater opportunity for developing a more complete biological insight. For example, since a positive value of $\log\left(\frac{a}{b}\right)$ indicates *a* is more abundant than *b*, a positive association of a phenotype with $\log\left(\frac{a}{b}\right)$ indicates that it is the increase in taxon *a* *relative to* taxon *b* that associates with the phenotype, not just the increase (decrease) in the count of taxon *a* (*b*) by itself. Finally, when used as summary statistics in between-group comparisons, multivariate logratio signatures extracted by selEnergyPerm are not limited to single taxon comparisons alone but may instead represent complex relative differential abundance patterns between mutliple taxa. Using logratio networks may additionally enable researchers to visualize and examine relationships between many taxon simultaneously.

Overall, our results demonstrate that SelEnergyPerm is a powerful framework for detecting sparse association under various scenarios. However, in the presence of heterogeneity of variance and/or unbalanced group designs—both of which are common enemies of multivariate association testing methods—the power of SelEnergyPerm was reduced, albeit to a lesser degree than the standard methods tested. Therefore, caution should be used when applying SelEnergyPerm in these settings. Additionally, in some scenarios with dense association signals, the performance of SelEnergyPerm was slightly reduced when compared to standard methods. While the power reduction was small, the enhanced interpretation from a smaller logratio signature may nevertheless outweigh the loss of power in such settings.

Notwithstanding these limitations, SelEnergyPerm is the first method to our knowledge to fully utilize the pairwise logratio compositional approach in a group association testing framework for metagenomic data. Importantly, given the compositional sample space imposed on these data, where features are relative, our approach enables the discovery of associations using pairwise logratios which, by design, robustly interpret features relative to one another rather than alone. While the benefits of employing logratios are well documented, implementing and carrying out these analyses using pairwise logratios can be challenging and time consuming in practice. To this end, we developed an R package, SelEnergyPermR, with functions to perform the method as developed and including the demonstrations utilized in this paper. Additionally, our package enables rapid preprocessing of relative abundance data, calculation of all pairwise logratios, and multiplicative zero imputation. Our package also includes functions to simulate data from all scenarios presented in this work. Lastly, our approach adds to a small list of compositional methods for testing associations (Fernandes et al., 2014; Mandal et al., 2015; Lin and Peddada, 2020) and is to our knowledge, the first compositional data method developed for sparse multivariate group association testing in metagenomic data. We also add to a small list of compositional approaches for feature selection (Susin et al., 2020); however, unlike these other methods, our approach directly uses pairwise logratios which enables simple interpretation and may better elucidate taxa-taxa interactions through logratio network analysis. While not demonstrated explicitly here, SelEnergyPerm is also compatible with multi-class (> 2 groups) group association testing (as implemented in our R package and demonstrated in Hickman et al., 2021). Future directions to usefully expand this methodology could focus on incorporating covariate information and extending the framework to longitudinal data.

## 5. Conclusion

We developed SelEnergyPerm to be a versatile group association testing method for detecting and understanding sparse associations in high-dimensional metagenomic data. We showed through rigorous simulation study with synthetic and real data distributions that SelEnergyPerm selects parsimonious subsets of independent logratios that better maximize between-group associations when compared to existing feature selection methods. In comparison to popular alternatives, we show the SelEnergyPerm feature selection approach is able to select fewer logratios, guarantee logratio subsets are independent, and better maximize between-group associations with relatively modest computational time requirements. To this end, our simulation results demonstrate SelEnergyPerm is significantly better at detecting sparse associations when compared to existing multivariate group association tests. Overall, SelEnergyPerm will enable researchers to robustly detect, characterize, and understand sparse associations in metagenomic data using novel logratio signatures. The SelEnergyPerm method is implemented in the R package SelEnergyPermR, freely available on GitHub (https://github.com/andrew84830813/selEnergyPermR.git), including an example demonstration and code for each of the analyses using the method presented here.

## Data Availability Statement

The original contributions presented in the study are included in the article/Supplementary Material, further inquiries can be directed to the corresponding author.

## Author Contributions

AH developed the framework methodology, performed simulations and case studies, and developed the software package. PM oversaw the development of the framework methodology. AH and PM together wrote the manuscript and approved the final manuscript. All authors contributed to the article and approved the submitted version.

## Funding

This research was funded by a Howard Hughes Medical Institute Gilliam Award (GT11504) and a James S. McDonnell Foundation Complex Systems Scholar Award (#220020315).

## Author Disclaimer

The content is solely the responsibility of the authors and does not necessarily represent the official views of any agency funding this research.

## Conflict of Interest

The authors declare that the research was conducted in the absence of any commercial or financial relationships that could be construed as a potential conflict of interest.

## Publisher's Note

All claims expressed in this article are solely those of the authors and do not necessarily represent those of their affiliated organizations, or those of the publisher, the editors and the reviewers. Any product that may be evaluated in this article, or claim that may be made by its manufacturer, is not guaranteed or endorsed by the publisher.

## References

[B1] Aitchison J. (1982). The statistical analysis of compositional data. J. R. Stat. Soc. B 44, 139–177. 10.1111/j.2517-6161.1982.tb01195.x

[B2] Anderson M. J. (2006). Distance-based tests for homogeneity of multivariate dispersions. Biometrics 62, 245–253. 10.1111/j.1541-0420.2005.00440.x 16542252

[B3] Anderson M. J. (2017). “Permutational multivariate analysis of variance (PERMANOVA),” in Wiley StatsRef: Statistics Reference Online (American Cancer Society), 1–15.

[B4] Anderson M. J. Walsh D. C. I. (2013). PERMANOVA, ANOSIM, and the Mantel test in the face of heterogeneous dispersions: what null hypothesis are you testing? Ecol. Monogr. 83, 557–574. 10.1890/12-2010.1

[B5] Baumann K. (2003). Cross-validation as the objective function for variable-selection techniques. Trends Anal. Chem. 22, 395–406. 10.1016/S0165-9936(03)00607-1

[B6] Bokulich N. A. Chung J. Battaglia T. Henderson N. Jay M. Li H. . (2016). Antibiotics, birth mode, and diet shape microbiome maturation during early life. Sci. Transl. Med. 8, 343ra82–343ra82. 10.1126/scitranslmed.aad7121 27306664 PMC5308924

[B7] Calgaro M. Romualdi C. Waldron L. Risso D. Vitulo N. (2020). Assessment of statistical methods from single cell, bulk RNA-seq, and metagenomics applied to microbiome data. Genome Biol. 21, 191. 10.1186/s13059-020-02104-1 32746888 PMC7398076

[B8] Caporaso J. G. Kuczynski J. Stombaugh J. Bittinger K. Bushman F. D. Costello E. K. . (2010). QIIME allows analysis of high-throughput community sequencing data. Nat. Methods 7, 335–336. 10.1038/nmeth.f.303 20383131 PMC3156573

[B9] Clarke K. R. (1993). Non-parametric multivariate analyses of changes in community structure. Austr. J. Ecol. 18, 117–143. 10.1111/j.1442-9993.1993.tb00438.x

[B10] Csardi G. Nepusz T. (2006). The igraph software package for complex network research. Inter. J. Compl. Syst. 1695, 1–9. 10.5281/zenodo.363026831819800

[B11] Dewhirst F. E. Chen T. Izard J. Paster B. J. Tanner A. C. R. Yu W.-H. . (2010). The human oral microbiome. J. Bacteriol. 192, 5002–5017. 10.1128/JB.00542-10 20656903 PMC2944498

[B12] Duvallet C. Gibbons S. Gurry T. Irizarry R. Alm E. (2017). MicrobiomeHD: the human gut microbiome in health and disease. Type: dataset. Zenodo. 10.5281/zenodo.569601

[B13] Egozcue J. J. Pawlowsky-Glahn V. Mateu-Figueras G. Barceló-Vidal C. (2003). Isometric logratio transformations for compositional data analysis. Math. Geol. 35, 279–300. 10.1023/A:1023818214614

[B14] Ernst M. D. (2004). Permutation methods: a basis for exact inference. Stat. Sci. 19, 676–685. 10.1214/088342304000000396

[B15] Fan J. Lv J. (2008). Sure independence screening for ultrahigh dimensional feature space. J. R. Stat. Soc. B 70, 849–911. 10.1111/j.1467-9868.2008.00674.x 19603084 PMC2709408

[B16] Fernandes A. D. Reid J. N. Macklaim J. M. McMurrough T. A. Edgell D. R. Gloor G. B. (2014). Unifying the analysis of high-throughput sequencing datasets: characterizing RNA-seq, 16S rRNA gene sequencing and selective growth experiments by compositional data analysis. Microbiome 2, 15. 10.1186/2049-2618-2-15 24910773 PMC4030730

[B17] Foster J. A. McVey Neufeld K.-A. (2013). Gut–brain axis: how the microbiome influences anxiety and depression. Trends Neurosci. 36, 305–312. 10.1016/j.tins.2013.01.005 23384445

[B18] Gloor G. B. Macklaim J. M. Pawlowsky-Glahn V. Egozcue J. J. (2017). Microbiome datasets are compositional: and this is not optional. Front. Microbiol. 8, 2224. 10.3389/fmicb.2017.02224 29187837 PMC5695134

[B19] Gloor G. B. Reid G. (2016). Compositional analysis: a valid approach to analyze microbiome high-throughput sequencing data. Can. J. Microbiol. 62, 692–703. 10.1139/cjm-2015-0821 27314511

[B20] Gonzalez A. Navas-Molina J. A. Kosciolek T. McDonald D. Vázquez-Baeza Y. Ackermann G. . (2018). Qiita: rapid, web-enabled microbiome meta-analysis. Nat. Methods 15, 796–798. 10.1038/s41592-018-0141-9 30275573 PMC6235622

[B21] Goodrich J. Waters J. Poole A. Sutter J. Koren O. Blekhman R. . (2014). Human genetics shape the gut microbiome. Cell 159, 789–799. 10.1016/j.cell.2014.09.053 25417156 PMC4255478

[B22] Gopalakrishnan V. Helmink B. A. Spencer C. N. Reuben A. Wargo J. A. (2018). The influence of the gut microbiome on cancer, immunity, and cancer immunotherapy. Cancer Cell 33, 570–580. 10.1016/j.ccell.2018.03.015 29634945 PMC6529202

[B23] Granitto P. M. Furlanello C. Biasioli F. Gasperi F. (2006). Recursive feature elimination with random forest for PTR-MS analysis of agroindustrial products. Chem. Intell. Lab. Syst. 83, 83–90. 10.1016/j.chemolab.2006.01.007

[B24] Greenacre M. (2019). Variable selection in compositional data analysis using pairwise logratios. Math. Geosci. 51, 649–682. 10.1007/s11004-018-9754-x34737726

[B25] Greenacre M. Lewi P. (2009). Distributional equivalence and subcompositional coherence in the analysis of compositional data, contingency tables and ratio-scale measurements. J. Classificat. 26, 29–54. 10.1007/s00357-009-9027-y

[B26] Hickman E. Hinton A. Zorn B. Rebuli M. Robinette C. Wolfgang M. . (2021). E-cigarette use, cigarette use, and sex modify the nasal microbiome and nasal host-microbiota interactions. 10.21203/rs.3.rs-725763/v2

[B27] Kent J. T. (1983). Information gain and a general measure of correlation. Biometrika 70, 163–173. 10.1093/biomet/70.1.163

[B28] Koh H. Zhao N. (2020). A powerful microbial group association test based on the higher criticism analysis for sparse microbial association signals. Microbiome 8, 63. 10.1186/s40168-020-00834-9 32393397 PMC7216722

[B29] Kong H. H. Oh J. Deming C. Conlan S. Grice E. A. Beatson M. A. . (2012). Temporal shifts in the skin microbiome associated with disease flares and treatment in children with atopic dermatitis. Genome Res. 22, 850–859. 10.1101/gr.131029.111 22310478 PMC3337431

[B30] Kuhn M. (2021). caret: Classification and Regression Training. R package version 6. 0–88.

[B31] Kursa M. B. Jankowski A. Rudnicki W. R. (2010). Boruta–a system for feature selection. Fundamenta Inf. 101, 271–285. 10.3233/FI-2010-288

[B32] Kursa M. B. Rudnicki W. R. (2010). Feature selection with the Boruta package. J. Stat. Softw. 36, 1–13. 10.18637/jss.v036.i11

[B33] Layeghifard M. Hwang D. M. Guttman D. S. (2017). Disentangling interactions in the microbiome: a network perspective. Trends Microbiol. 25, 217–228. 10.1016/j.tim.2016.11.008 27916383 PMC7172547

[B34] Lin H. Peddada S. D. (2020). Analysis of compositions of microbiomes with bias correction. Nat. Commun. 11, 3514. 10.1038/s41467-020-17041-7 32665548 PMC7360769

[B35] Lindgren F. Hansen B. Karcher W. Sjöström M. Eriksson L. (1996). Model validation by permutation tests: applications to variable selection. J. Chemometr. 10, 521–532. 10.1002/(SICI)1099-128X(199609)10:5/6<521::AID-CEM448>3.0.CO;2-J

[B36] Lloyd-Price J. Arze C. Ananthakrishnan A. N. Schirmer M. Avila-Pacheco J. Poon T. W. . (2019). Multi-omics of the gut microbial ecosystem in inflammatory bowel diseases. Nature 569, 655–662. 10.1038/s41586-019-1237-9 31142855 PMC6650278

[B37] Lovell D. R. Chua X.-Y. McGrath A. (2020). Counts: an outstanding challenge for log-ratio analysis of compositional data in the molecular biosciences. NAR Genom. Bioinformatics 2, lqaa040. 10.1093/nargab/lqaa040 33575593 PMC7671413

[B38] Mandal S. Treuren W. V. White R. A. Eggesbø M. Knight R. Peddada S. D. (2015). Analysis of composition of microbiomes: a novel method for studying microbial composition. Microb. Ecol. Health Dis. 26, 27663. 10.3402/mehd.v26.27663 26028277 PMC4450248

[B39] Manichanh C. Borruel N. Casellas F. Guarner F. (2012). The gut microbiota in IBD. Nat. Rev. Gastroenterol. Hepatol. 9, 599–608. 10.1038/nrgastro.2012.152 22907164

[B40] Martín R. Miquel S. Langella P. Bermúdez-Humarán L. G. (2014). The role of metagenomics in understanding the human microbiome in health and disease. Virulence 5, 413–423. 10.4161/viru.27864 24429972 PMC3979869

[B41] Martın-Fernández J.-A. Hron K. Templ M. Filzmoser P. Palarea-Albaladejo J. (2015). Bayesian-multiplicative treatment of count zeros in compositional data sets. Stat Model. 15, 134–158. 10.1177/1471082X14535524

[B42] Martın-Fernandez J. A. Barcelo-Vidal C. Pawlowsky-Glahn V. (2003). Dealing with zeros and missing values in compositional data sets using nonparametric imputation. Math. Geol. 26, 273–278. 10.1023/A:1023866030544

[B43] Microsoft and Weston S.. (2020). foreach: Provides Foreach Looping Construct. R package version 1.5.1.

[B44] Obuchowski N. A. (2005). Multivariate statistical methods. Am. J. Roentgenol. 185, 299–309. 10.2214/ajr.185.2.01850299 16037496

[B45] Oksanen J. Blanchet F. G. Friendly M. Kindt R. Legendre P. McGlinn D. . (2020). vegan: Community Ecology Package. R package version 2. 5–7.

[B46] Oliveira F. S. Brestelli J. Cade S. Zheng J. Iodice J. Fischer S. . (2018). MicrobiomeDB: a systems biology platform for integrating, mining and analyzing microbiome experiments. Nucleic Acids Res. 46, D684–D691. 10.1093/nar/gkx1027 29106667 PMC5753346

[B47] Palarea-Albaladejo J. Martín-Fernández J. A. (2015). zCompositions—R package for multivariate imputation of left-censored data under a compositional approach. Chemometr. Intell. Lab. Syst. 143:85–96. 10.1016/j.chemolab.2015.02.019

[B48] Pasolli E. Schiffer L. Manghi P. Renson A. Obenchain V. Truong D. T. . (2017). Accessible, curated metagenomic data through ExperimentHub. Nat. Methods 14, 1023–1024. 10.1038/nmeth.4468 29088129 PMC5862039

[B49] Paulson J. N. Williams B. L. Hehnly C. Mishra N. Sinnar S. A. Zhang L. . (2020). Paenibacillus infection with frequent viral coinfection contributes to postinfectious hydrocephalus in Ugandan infants. Sci. Transl. Med. 12, eaba0565. 10.1126/scitranslmed.aba0565 32998967 PMC7774825

[B50] Pawlowsky-Glahn V. Buccianti A. (2011). Compositional Data Analysis: Theory and Applications. London: John Wiley & Sons.

[B51] Pearson K. (1897). Mathematical contributions to the theory of evolution.—On a form of spurious correlation which may arise when indices are used in the measurement of organs. Proc R. Soc. Lond. 60, 489–498. 10.1098/rspl.1896.0076

[B52] Proctor L. M. Creasy H. H. Fettweis J. M. Lloyd-Price J. Mahurkar A. Zhou W. . (2019). The integrative human microbiome project. Nature 569, 641–648. 10.1038/s41586-019-1238-8 31142853 PMC6784865

[B53] Qin N. Yang F. Li A. Prifti E. Chen Y. Shao L. . (2014). Alterations of the human gut microbiome in liver cirrhosis. Nature 513, 59–64. 10.1038/nature13568 25079328

[B54] Quinn T. P. Erb I. Gloor G. Notredame C. Richardson M. F. Crowley T. M. (2019). A field guide for the compositional analysis of any-omics data. GigaScience 8, giz107. 10.1093/gigascience/giz107 31544212 PMC6755255

[B55] Ranjan R. Rani A. Metwally A. McGee H. S. Perkins D. L. (2016). Analysis of the microbiome: advantages of whole genome shotgun versus 16S amplicon sequencing. Biochem. Biophys. Res. Commun. 469, 967–977. 10.1016/j.bbrc.2015.12.083 26718401 PMC4830092

[B56] Risso D. Perraudeau F. Gribkova S. Dudoit S. Vert J.-P. (2018). A general and flexible method for signal extraction from single-cell RNA-seq data. Nat. Commun. 9:284. 10.1038/s41467-017-02554-5 29348443 PMC5773593

[B57] Rizzo M. Szekely G. (2021). energy: E-Statistics: Multivariate Inference via the Energy of Data. R package version 1.7–8.

[B58] Rizzo M. L. Székely G. J. (2010). DISCO analysis: a nonparametric extension of analysis of variance. Ann. Appl. Stat. 4, 1034–1055. 10.1214/09-AOAS245

[B59] Rizzo M. L. Székely G. J. (2016). Energy distance. Wiley Interdisc. Rev. 8, 27–38. 10.1002/wics.1375

[B60] Schlaberg R. (2020). Microbiome diagnostics. Clin. Chem. 66, 68–76. 10.1373/clinchem.2019.303248 31843867

[B61] Schloss Patrick D. Westcott Sarah L. Ryabin T.homas Hall Justine R. Hartmann M. artin Hollister Emily B. Lesniewski Ryan A. Oakley Brian B. . (2009). Introducing mothur: open-source, platform-independent, community-supported software for describing and comparing microbial communities. Appl. Environ. Microbiol. 75, 7537–7541. 10.1128/AEM.01541-09 19801464 PMC2786419

[B62] Simon N. Friedman J. Hastie T. Tibshirani R. (2011). Regularization paths for cox's proportional hazards model via coordinate descent. J. Stat. Softw. 39, 1–13. 10.18637/jss.v039.i05 27065756 PMC4824408

[B63] Susin A. Wang Y. Lê Cao K.-A. Calle M. L. (2020). Variable selection in microbiome compositional data analysis. NAR Genomics Bioinformatics 2, lqaa029. 10.1093/nargab/lqaa029 33575585 PMC7671404

[B64] Tibshirani R. (1996). Regression shrinkage and selection via the lasso. J. R. Stat. Soc. B 58, 267–288. 10.1111/j.2517-6161.1996.tb02080.x

[B65] Truong D. T. Franzosa E. A. Tickle T. L. Scholz M. Weingart G. Pasolli E. . (2015). MetaPhlAn2 for enhanced metagenomic taxonomic profiling. Nat. Methods 12, 902–903. 10.1038/nmeth.3589 26418763

[B66] Vatanen T. Kostic A. D. d'Hennezel E. Siljander H. Franzosa E. A. Yassour M. . (2016). Variation in microbiome LPS immunogenicity contributes to autoimmunity in humans. Cell 165, 842–853. 10.1016/j.cell.2016.04.007 27133167 PMC4950857

[B67] Wei S. Lee C. Wichers L. Marron J. S. (2016). Direction-projection-permutation for high-dimensional hypothesis tests. J. Comput. Graph. Stat. 25, 549–569. 10.1080/10618600.2015.1027773

[B68] Weiss S. Xu Z. Z. Peddada S. Amir A. Bittinger K. Gonzalez A. . (2017). Normalization and microbial differential abundance strategies depend upon data characteristics. Microbiome 5, 27. 10.1186/s40168-017-0237-y 28253908 PMC5335496

[B69] Wilson M. T. Hamilos D. L. (2014). The nasal and sinus microbiome in health and disease. Curr. Allergy Asthma Rep. 14, 485. 10.1007/s11882-014-0485-x 25342392

[B70] Wood D. E. Lu J. Langmead B. (2019). Improved metagenomic analysis with Kraken 2. Genome Biol. 20, 257. 10.1186/s13059-019-1891-0 31779668 PMC6883579

[B71] Wu C. Chen J. Kim J. Pan W. (2016). An adaptive association test for microbiome data. Genome Med. 8, 56. 10.1186/s13073-016-0302-3 27198579 PMC4872356

[B72] Zackular J. P. Rogers M. A. M. Ruffin M. T. Schloss P. D. (2014). The human gut microbiome as a screening tool for colorectal cancer. Cancer Prevent. Res. 7, 1112–1121. 10.1158/1940-6207.CAPR-14-0129 25104642 PMC4221363

[B73] Zawadzki Z. Kosinski M. (2021). FSelectorRcpp: 'Rcpp' Implementation of 'FSelector' Entropy-Based Feature Selection Algorithms with a Sparse Matrix Support. R package version 0.3.8.

[B74] Zeevi D. Korem T. Zmora N. Israeli D. Rothschild D. Weinberger A. . (2015). Personalized nutrition by prediction of glycemic responses. Cell 163, 1079–1094. 10.1016/j.cell.2015.11.001 26590418

