# Supplementary material for: A Simultaneous Feature Selection and Compositional Association Test for Detecting Sparse Associations in High-Dimensional Metagenomic Data
Source: Front Microbiol. 2022 Mar 21;13:837396. doi: 10.3389/fmicb.2022.837396 (PMC8978828; doi:10.3389/fmicb.2022.837396)
Supplement: Supplementary file 1 [file Data_Sheet_1.PDF]

Supplementary Material

1 PSEUDO CODE

Algorithm 1 Association maximization with greedy forward stepwise selection

```
procedure SELECTIONENERGY(Z', y, α, patience)
    if PERMDISP2(Z'y) < α then                          ▷ Determine test statistic
        testStatisticFunction = cF()
        Metric = 'combinedF'
    else
        testStatisticFunction = F_{n,α}()
        Metric = 'discoF'
    end if
    X = Z' ∈ ℝ^{n×3}                                     ▷ Select first 3 columns
    maxF = testStatisticFunction(baseSet,y)
    improvementTime = 0
    for i ∈ [4, . . . , |Z'|] do
        X_new = X ∪ z'_{*,i}                             ▷ Append ith column
        newF = testStatisticFunction(newSet,y)
        diff = newF - maxF
        if diff ≥ eps then
            X = X_new
            maxF = newF
            improvementTime = 0
        else
            improvementTime = improvementTime +1
        end if
        if improvementTime > patience then
            Break
        end if
    end for
    return(X , testStat )
end procedure
```

2 SUPPLEMENTARY FIGURES

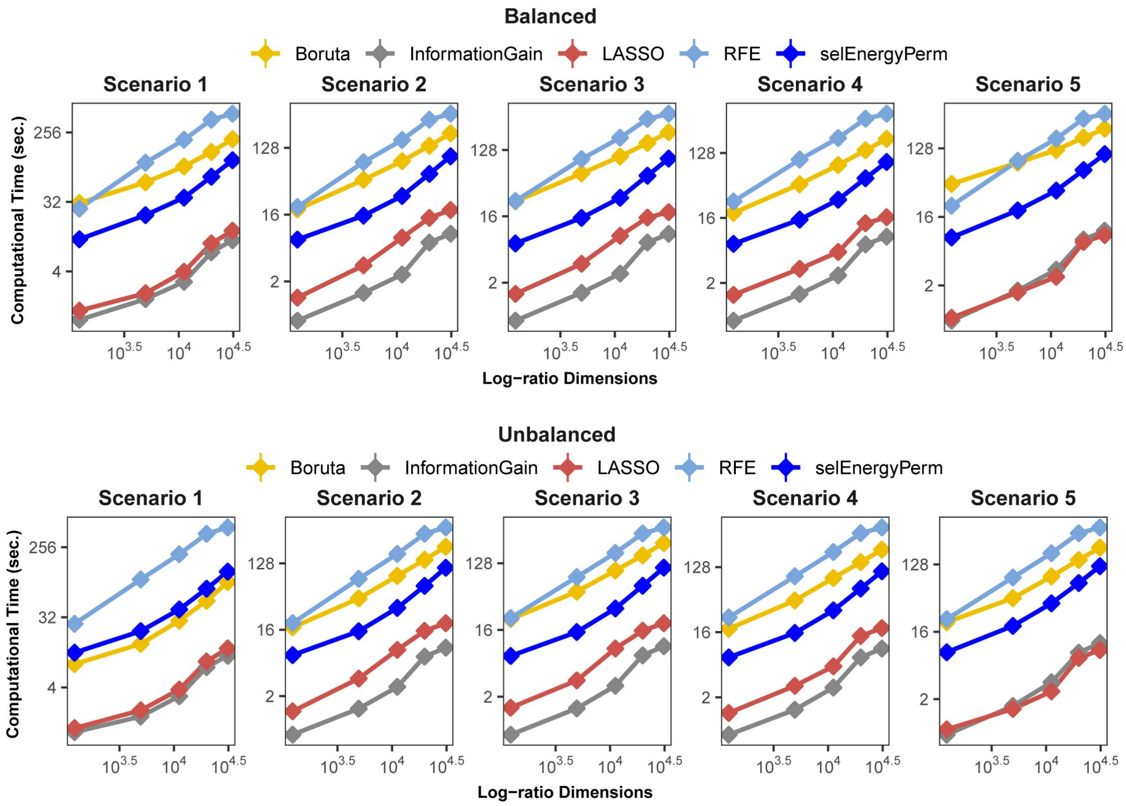

Figure S1. Feature selection computational time comparisons for balanced and unbalanced sampling designs between SelEnergyPerm, LASSO, RFE, RF, Information Gain, and Boruta across each scenario and dimension. Points are the mean for each experimental condition.

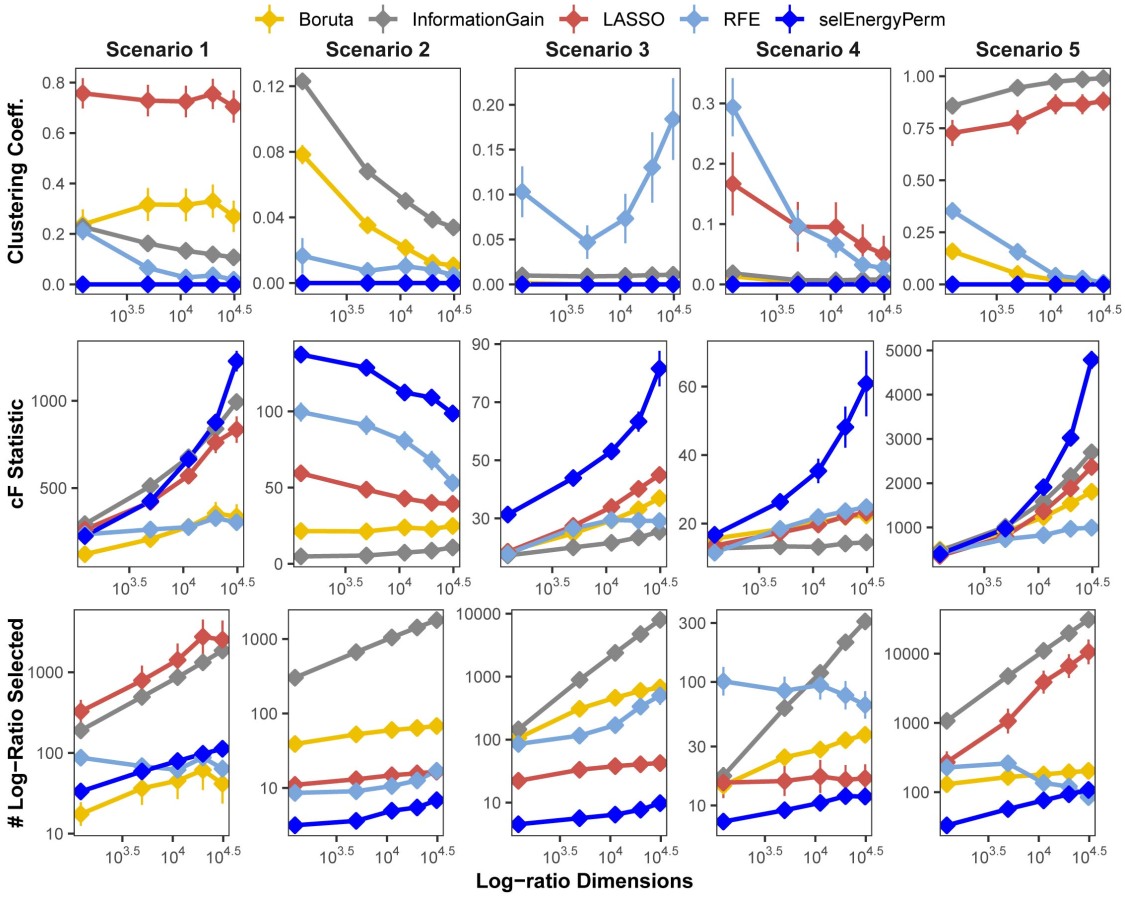

Figure S2. Comparison of SelEnergyPerm-selected log-ratio subset characteristics with Boruta, Information Gain Filtering, LASSO, and RFE across five simulation scenarios for the unbalanced sampling design. Using 200 simulations for each scenario-dimension by method we assessed: (Top Row) the clustering coefficient of log ratio networks formed by selected subsets returned from each method, (Middle Row) the magnitude of the association as measured by the cF-statistic on selected subsets returned from each method, and (Bottom Row) the number of log ratios returned by each method. Points are the mean for each experimental condition and error bars indicate 95% confidence interval.

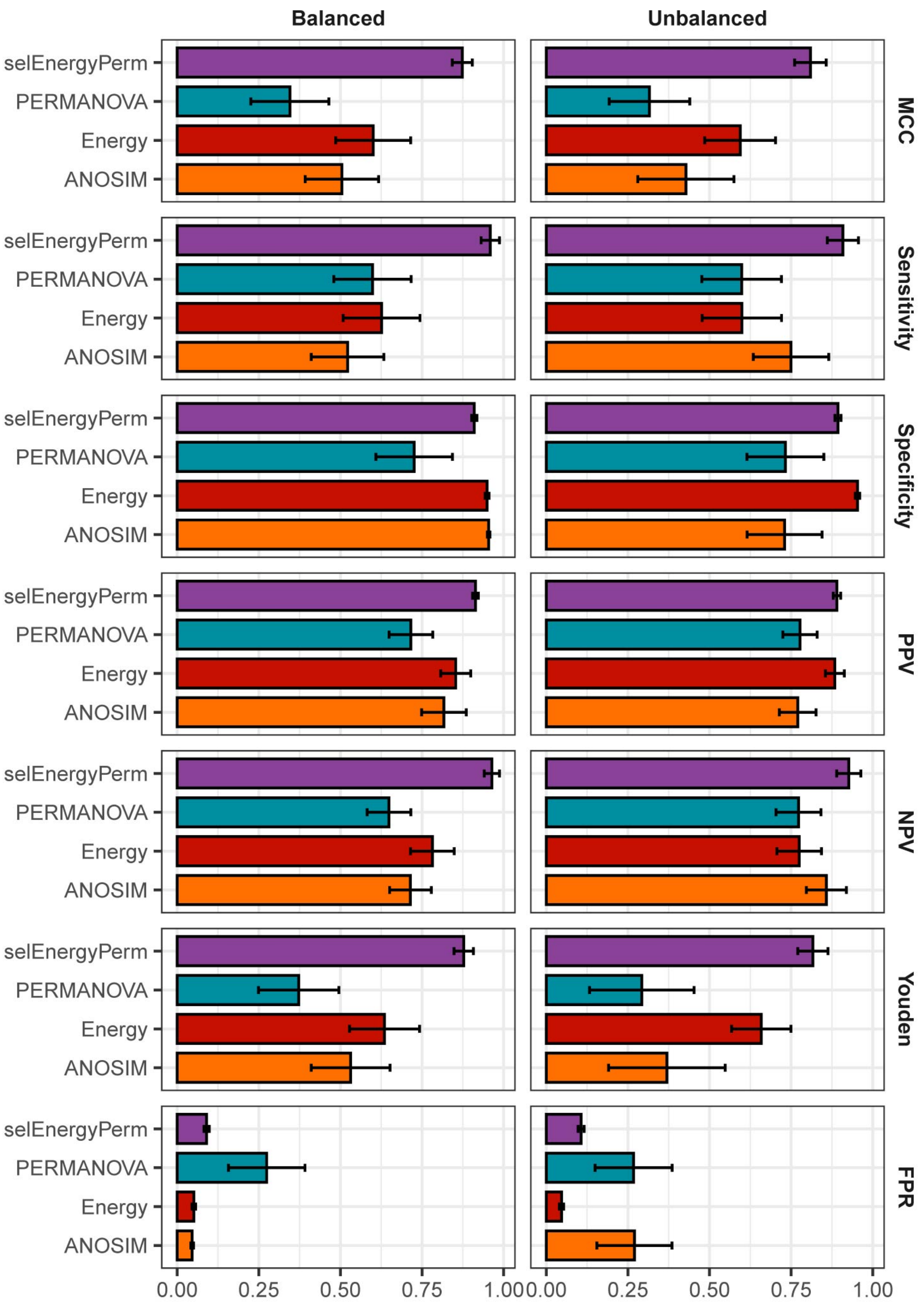

Figure S3. Overall mean performance comparison for data generated from synthetic distributions aggregated across all scenarios and dimensions using MCC, Sensitivity, Specify, Positive predictive value (PPV), Negative predictive value (NPV), Youden Index, and False Positive Rate (FPR) metric. Error bars indicate standard error.

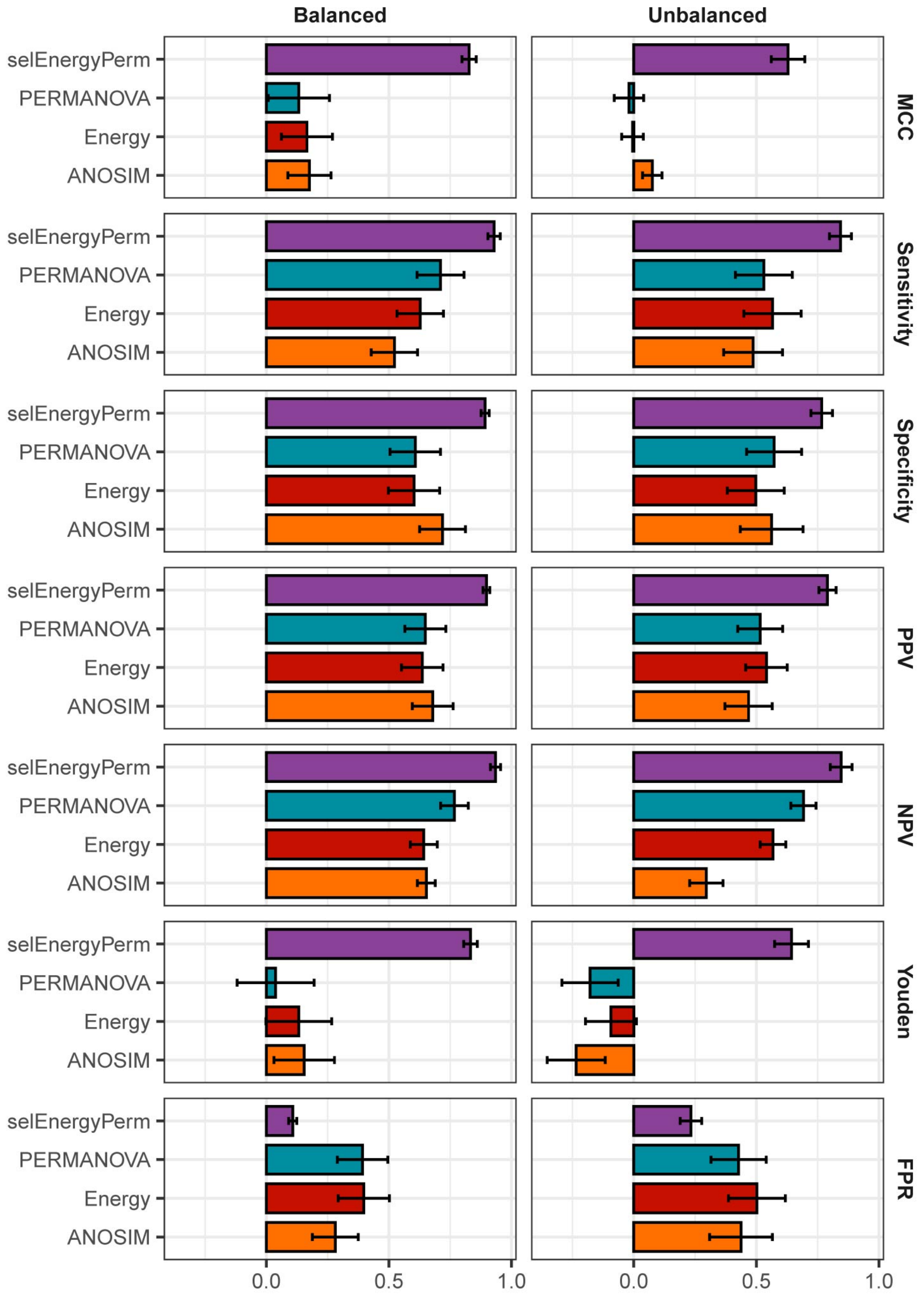

Figure S4. Overall mean performance comparison for data generated from 16S and WGS synthetic data aggregated across all scenarios and effect levels using MCC, Sensitivity, Specify, Positive predictive value (PPV), Negative predictive value (NPV), Youden Index, and False Positive Rate (FPR) metric. Error bars indicate standard error.
